# Altered Fast Synaptic Transmission in a Mouse Model of DNM1-Associated Developmental Epileptic Encephalopathy

**DOI:** 10.1523/ENEURO.0269-20.2020

**Published:** 2021-03-09

**Authors:** Matthew P. McCabe, Amy N. Shore, Wayne N. Frankel, Matthew C. Weston

**Affiliations:** 1 Department of Neurological Sciences, Larner College of Medicine, University of Vermont, Burlington, VT 05405; 2 Department of Genetics and Development, Institute for Genomic Medicine, Columbia University, New York, NY 10032

**Keywords:** cell death, dynamin, encephalopathy, epilepsy, network activity, synaptic transmission

## Abstract

Developmental epileptic encephalopathies (DEEs) are severe seizure disorders that occur in infants and young children, characterized by developmental delay, cognitive decline, and early mortality. Recent efforts have identified a wide variety of genetic variants that cause DEEs. Among these, variants in the *DNM1* gene have emerged as definitive causes of DEEs, including infantile spasms and Lennox–Gastaut syndrome. A mouse model of *Dnm1*-associated DEE, known as “Fitful” (*Dnm1^Ftfl^*), recapitulates key features of the disease, including spontaneous seizures, early lethality, and neuronal degeneration. Previous work showed that DNM1 is a key regulator of synaptic vesicle (SV) endocytosis and synaptic transmission and suggested that inhibitory neurotransmission may be more reliant on DNM1 function than excitatory transmission. The *Dnm1^Ftfl^* variant is thought to encode a dominant negative DNM1 protein; however, the effects of the *Dnm1^Ftfl^* variant on synaptic transmission are largely unknown. To understand these synaptic effects, we recorded from pairs of cultured mouse cortical neurons and characterized all four major connection types [excitation of excitation (E-E), inhibition of inhibition (I-I), E-I, I-E]. Miniature and spontaneous EPSCs and IPSCs were larger, but less frequent, at all *Dnm1^Ftfl^* synaptic types, and *Dnm1^Ftfl^* neurons had reduced expression of excitatory and inhibitory SV markers. Baseline evoked transmission, however, was reduced only at inhibitory synapses onto excitatory neurons, because of a smaller pool of releasable SVs. In addition to these synaptic alterations, *Dnm1^Ftfl^* neurons degenerated later in development, although their activity levels were reduced, suggesting that *Dnm1^Ftfl^* may impair synaptic transmission and neuronal health through distinct mechanisms.

## Significance Statement

Recent work has identified genetic variants in *DNM1* as among the more common causes of developmental epileptic encephalopathies (DEEs), but the physiological consequences of its mutation are unclear. Here, we make use of an *in vitro* model of *Dnm1*-associated DEE to determine the effects of a *Dnm1* variant on the four main cortical synapse types. The variant caused both synapse-specific and synapse-wide alterations, and decreased neuronal activity. Despite this, neurons still degenerated after two weeks *in vitro*, suggesting that neuronal degeneration in these mice may be independent of seizures, and that stopping seizures may do little to mitigate key features of DEEs.

## Introduction

Developmental epileptic encephalopathies (DEEs) are childhood disorders characterized by severe seizures, developmental delay, cognitive decline, and death. Recent work in DEE genetics has identified over 150 likely causative *de novo* gene variants in children with DEE (Epi4K Consortium, Epilepsy Phenome/Genome Project, 2013; EuroEPINOMICS-RES Consortium, Epilepsy Phenome/Genome Project, Epi4K Consortium, 2014). Many of these variants occur in genes either known or predicted to regulate synaptic transmission, suggesting synaptic dysfunction may be a common feature of DEE. One such gene, *DNM1*, has emerged as one of the most prevalent genetic causes of DEEs (EuroEPINOMICS-RES Consortium, Epilepsy Phenome/Genome Project, Epi4K Consortium, 2014; von Spiczak et al., 2017). Children with pathologic *DNM1* variants have severe seizures and experience cognitive decline, typically presenting with infantile spasms that progress to Lennox–Gastaut syndrome (von Spiczak et al., 2017). Developmental delay generally begins in the first year of life, often before seizure onset (von Spiczak et al., 2017). In some cases, cerebral atrophy or defects in myelination of the corpus callosum can be seen on MRI scans by two years of age, suggesting children with *DNM1* variants undergo progressive degeneration (von Spiczak et al., 2017).

Basic research in mice indicates the *Dnm1* gene is primarily expressed in the central nervous system (Ferguson and De Camilli, 2012) and that its protein product, dynamin-1, is important for synaptic vesicle (SV) endocytosis (Ferguson et al., 2007; Ferguson and De Camilli, 2012). Knock-out of *Dnm1* revealed that it plays an activity-dependent role in maintaining the SV pool and synaptic transmission (Ferguson et al., 2007; Armbruster et al., 2013). *Dnm1* is also important for regulating SV size, as knock-out leads to larger, more heterogeneously sized SVs and miniature PSCs (Ferguson et al., 2007). Finally, *Dnm1* loss has a stronger effect on inhibitory synapses, as the increase in clathrin coat components that occurs as a result of *Dnm1* KO correlates more with vesicular GABA transporter (VGAT) expression than vesicular glutamate transporter (VGLUT) expression (Ferguson et al., 2007; Hayashi et al., 2008).

DNM1-associated DEE’s have been modeled in mice. Homozygous expression of a spontaneous variant in the mouse ortholog of *Dnm1*, *Dnm1^Ftfl^*, causes many of the disease features associated with human *DNM1* patients, including seizures, developmental decline between postnatal day (P)14 and P21, neuronal degeneration, (Aimiuwu et al., 2020) and death around weaning age (Boumil et al., 2010; Asinof et al., 2015; Aimiuwu et al., 2020). Previous work in *Dnm1^Ftfl/Ftfll^* mice suggests *Dnm1^Ftfl^* is a dominant-negative variant, and causes seizures and death through its action in inhibitory neurons. In particular, while *Dnm1^Ftfl/Ftfl^* mice have seizures and die, mice lacking exon 10a of the *Dnm1* gene, which harbors the *Dnm1^Ftfl^* variant, are viable and do not have obvious behavioral abnormalities (Asinof et al., 2016). Additionally, expression of *Dnm1^Ftfl^* in several subtypes of inhibitory neurons leads to seizures and death, while expression in excitatory cortical and hippocampal neurons produces behavioral abnormalities but not seizures (Asinof et al., 2015). Initial reports suggested *Dnm1^Ftfl^* also leads to enhanced depression of inhibitory synapses (Boumil et al., 2010), but the effects of *Dnm1^Ftfl^* on key aspects of synaptic transmission remain largely unknown.

To better understand whether *Dnm1^Ftfl^* has specific effects on synaptic inhibition, we recorded from pairs of cultured mouse cortical neurons and assessed synaptic function at the four main cortical synaptic motifs: excitatory synapses onto excitatory (glutamatergic) neurons [excitation of excitation (E-E)], inhibitory synapses onto inhibitory (GABAergic) neurons [inhibition of inhibition (I-I)], and mixed pairs (E-I and I-E). Many of the synaptic changes caused by *Dnm1^Ftfl^*, such as increased size and decreased frequency of miniature EPSCs (mEPSCs) and miniature IPSCs (mIPSCs) were observed at all four motifs. Additionally, markers of both excitatory and inhibitory SVs were reduced. However, baseline evoked PSCs (ePSCs) were only affected at the I-E motif, because of a smaller pool of readily releasable SVs. Despite these synaptic effects, *Dnm1^Ftfl^* cultures showed reductions in overall activity, and yet, *Dnm1^Ftfl^* neurons degenerate after two weeks *in vitro* [days *in vitro* (DIV)], suggesting that *Dnm1^Ftfl^* may impair neuronal health, leading to degeneration, through mechanisms other than seizures or hyperactivity.

## Materials and Methods

### Mice and cell culture

*Dnm1^Ftfl^* (Fitful) mice have been previously described (Boumil et al., 2010). All animal housing and care was in compliance with the National Institutes of Health *Guide for the Care and Use of Laboratory Animals.* All animal procedures were performed in accordance with the University of Vermont animal care committee’s regulations.

Astrocyte culture preparation began with coating of 23-mm diameter coverslips in a collagen and poly-d-lysine solution. Astrocytes derived from cerebral cortices of wild-type (WT) mice of either sex were plated onto these coverslips and allowed to achieve confluence, typically around one week. For primary neuron cultures, cerebral cortices of homozygous Fitful mice and WT littermate controls were microdissected from P0–P1 mice of either sex. Cortices were then digested with papain (Worthington) for 60 min and dissociated into cell suspensions. Neurons were plated at a density of 2–2.5 × 10^5^ neurons per well in six-well plates containing Neurobasal-A medium (NBA, Thermo Fisher Scientific) supplemented with B-27 and Glutamax (Invitrogen). When noted, each well was treated with one of either 1 μl of rAAV8-CaMKIIa-eGFP (UNC Vector Core) or 0.3 μl of pAAV8-mDlx-NLS-mRuby2 (Addgene) to allow for targeted patch-clamp of specific motifs. For calcium imaging experiments, each well was treated with 1 μl of AAV9-CaMKII-GCaMP6f (UPenn Vector Core).

### Calcium imaging

Cultures were transferred from incubators to the imaging stage of an inverted Olympus IX73 epifluorescent microscope with an Olympus PlanFluor 10× objective lens. Images were acquired using an Andor Zyla sCMOS 4.2 camera controlled by μManager 2.0-β (Edelstein et al., 2014). Images were acquired at a rate of 25 frames per second for 20 min. Fluorescent light intensity was set manually each day using control cultures to optimize the signal-to-noise ratio. Images were processed using ImageJ (Schindelin et al., 2012). Regions of interest (ROIs) were drawn manually using fluorescence changes and morphology to identify individual neurons, and fluorescence traces were then extracted. Postprocessing was conducted in Python. Change in fluorescence relative to baseline (ΔF/F) was calculated using a 1-min rolling baseline. The lower 10th percentile of the rolling window was used as the baseline fluorescence. ΔF/F traces were then used to detect fluorescence events using a derivative threshold.

### Electrophysiology

Whole-cell voltage-clamp recordings were performed with a patch-clamp amplifier (MultiClamp 700B amplifier; Molecular Devices) controlled by Clampex 10.7 (Molecular Devices, pClamp). Data were acquired at 10 kHz and low-pass filtered at 4 kHz. Series resistance was compensated at 70% and only cells with series resistance of <20 MΩ and stable holding currents of <500 pA were analyzed. Pipette resistance was between 2–5 MΩ. Extracellular solution contained the following: 140 mm NaCl, 2.4 mm KCl, 10 mm HEPES, 10 mm glucose, 4 mm MgCl_2_, and 2 mm CaCl_2_, pH 7.3 (300 mOsm). Internal solution contained: 136 mm KCl, 17.8 mm HEPES, 1 mm EGTA, 0.6 mm MgCl2,4 mm ATP, 0.3 mm GTP, 12 mm creatine phosphate, and 50 U/ml phosphocreatine kinase. Because of these concentrations, the chloride reversal potential allowed for detection of GABAergic synaptic responses as inward currents. All experiments were performed at room temperature (22–23°C). Recordings were taken from both Fitful and WT neurons on the same day (days 11–16 *in vitro* (DIV)). In voltage-clamp experiments, neurons were held at −70 mV.

For paired recordings, two nearby neurons were selected on the basis of the motif being targeted and the fluorescence of the neurons (for example, in experiments where the I-E motif was targeted and the rAAV8-CaMKIIa-eGFP virus was used, one eGFP+ neuron and one eGFP– neuron were assessed). Action potentials (APs) were evoked via 2-ms somatic depolarization to 0 mV at a rate of 0.1 Hz to assess baseline evoked EPSCs and IPSCs. From connected pairs, the neurotransmitter profile was assessed by a combination of decay time and application of either kynurenic acid (Kyn; 3 mm; Tocris) or NBQX (10 μm, Tocris) to block excitatory synaptic currents, or bicuculline (Bic; 20 μm; Tocris) to block inhibitory synaptic currents. The size of the readily-releasable pool (RRP) was assessed as previously described (Kaeser and Regehr, 2017). In brief, 100-Hz stimulation was applied to presynaptic inhibitory neurons. The postsynaptic response was integrated from baseline, and this integrated charge was plotted as a cumulative sum across stimuli. A line was fit to the linear phase of the corresponding cumulative charge plot and extrapolated back to the *y*-axis. The *y*-intercept was then used as an estimate of the amount of charge contained in the RRP. The charge evoked by the first stimulus was divided by the charge contained in the RRP to obtain the release probability. All analyses were conducted using Stimfit (Guzman et al., 2014).

For cell-attached recordings, the external solution was the same as that used for whole-cell recordings, except MgCl_2_ and CaCl_2_ were reduced to 1 and 2 mm, respectively, and KCl was increased to 5.4 mm, to better reflect the ionic concentration of the cell culture media. Pipettes were filled with the same external solution. After seal formation in voltage-clamp mode at a holding potential of −70 mV, the command potential was adjusted to achieve zero current flow. After a stable baseline was achieved, 200 s of current data were recorded at a sampling rate of 20 kHz. Traces were then exported to AxoGraph and events corresponding to APs were identified with a threshold crossing detection method. Timestamps were then exported to SPSS for further analysis.

### Spontaneous and miniature event detection

Spontaneous and miniature PSCs were obtained using similar methodologies. Spontaneous synaptic events were recorded for 60–80 s from paired neurons in 3 mm Kyn or 20 μm Bic to isolate inhibitory and excitatory sPSCs, respectively. mPSCs were recorded from individual neurons for 60–120 s in either Kyn or Bic, as well as 500 nm tetrodotoxin (TTX; Tocris) to block AP-generated synaptic events. For each cell, data were filtered at 1 kHz and analyzed using an optimally scaled sliding template-based detection algorithm (Clements and Bekkers, 1997) implemented in Stimfit analysis software (Guzman et al., 2014).

### Immunocytochemistry

Neurons were patch clamped using a modified internal solution containing 0.2% Biocytin (Sigma). Following recording, coverslips were transferred directly into 4% PFA for 30 min, washed with PBS three times for 5 min each, and then stored at 4°C in PBS 1–5 d. Coverslips were then placed in a blocking solution (10% NGS, 0.1% Triton X-100, and PBS) for 1 h at room temperature. Primary antibodies suspended in blocking solution were applied to coverslips overnight at 4°C. Primary antibodies used were VGLUT1 (mouse monoclonal, 1:5000 dilution, Synaptic Systems, catalog #135011) and VGAT (guinea pig polyclonal, 1:5000 dilution, Synaptic Systems, catalog #131004). Following primary antibody application, coverslips were washed three times with PBS and then secondary antibodies suspended in PBS were applied for 1 h at room temperature. Secondary antibodies were Alexa Fluor 488 goat anti-mouse (Invitrogen, 1:1000 dilution, used with the pAAV8-mDlx-NLS-mRuby2 virus), Alexa Fluor 594 goat anti-mouse (Invitrogen, 1:1000 dilution, used with the rAAV8-CaMKIIa-eGFP virus), Alexa Fluor 647 goat anti-guinea pig (Invitrogen, 1:1000 dilution), and Alexa Fluor 405 streptavidin (Invitrogen, 1:1000 dilution). Coverslips were then rinsed three times in PBS and mounted to slides with Prolong Gold Antifade (Invitrogen).

### Synapse imaging and analysis

Images (1024 × 1024 pixels) were obtained using a Nikon Ti-E Inverted Microscope with C2 Confocal using a 60× oil-immersion objective lens. Images were acquired with equal exposure times in stacks of 8–12 images obtained with 2× frame averaging to reduce background noise. Stacks were processed in Fiji software (Schindelin et al., 2012) to make maximum intensity projections. Synapses were then counted and cell area estimated using maximum intensity projections in the automated Intellicount software (Fantuzzo et al., 2017). For quantification of somatic synapse counts, the somatic region was defined manually, and synaptic puncta were counted manually. This value was then normalized to the total number of synapses.

### Cell death imaging and analysis

Coverslips were taken at DIV17 or DIV21 and transferred into extracellular solution (described above) supplemented with 50 μm propidium iodide (PI). Random representative fields of view were imaged on an inverted Olympus IX73 epifluorescent microscope with an Olympus PlanFluor 10× objective lens. Images were acquired using an Andor Zyla sCMOS 4.2 camera controlled by μManager 2.0-β (Edelstein et al., 2014). Fluorescent nuclei were then counted by two blind observers and their counts were averaged.

To quantify the density of excitatory and inhibitory neurons, each well was treated with 1 μl of rAAV8-CaMKIIa-eGFP (UNC Vector Core) and 0.2 μl of pAAV8-mDlx-NLS-mRuby2 (Addgene) at DIV3. On DIV10, DIV12, and DIV14, 10 live images (five eGFP and five mCherry) were collected from each group (three WT, three Dnm1^Ftfl^) using the same imaging set up used for Ca^2+^ imaging and the same acquisition parameters for each group.

### Statistics

All statistical analyses were performed using IBM SPSS version 26. Statistical significance was assessed using generalized estimating equations (GEEs). This approach has several advantages over other statistical methods both for analyzing our data and for answering our questions. First, it allows grouping of all observations together by accounting for between-culture differences. This prevents individual cultures with extreme effects from producing spuriously significant results, while avoiding the loss of power associated with reducing all observations from a culture to a single average. Second, as an extension of the generalized linear model, the GEE allows for ANOVA-like comparisons on data that is not normally distributed, which is the case for most synaptic data. These comparisons are necessary to directly test hypotheses about the generality and motif specificity of effects. Cultures were assigned unique identifiers and were used as the subject variable. For electrophysiological and fluorescence imaging assays either genotype alone or genotype and motif were used as model factors, and we used an exchangeable correlation matrix, model-based estimator, and modeled the data as γ distributed with log link. For calcium imaging assays, genotype and developmental epoch were used as factors, and we used an exchangeable correlation matrix, robust estimator, and modeled the data as either γ distributed (for peak fluorescence) or Poisson distributed (for event count) with log link.

## Results

### Miniature PSCs are larger but less frequent in *Dnm1^Ftfl/Ftfl^* cultures

**Figure 1.**
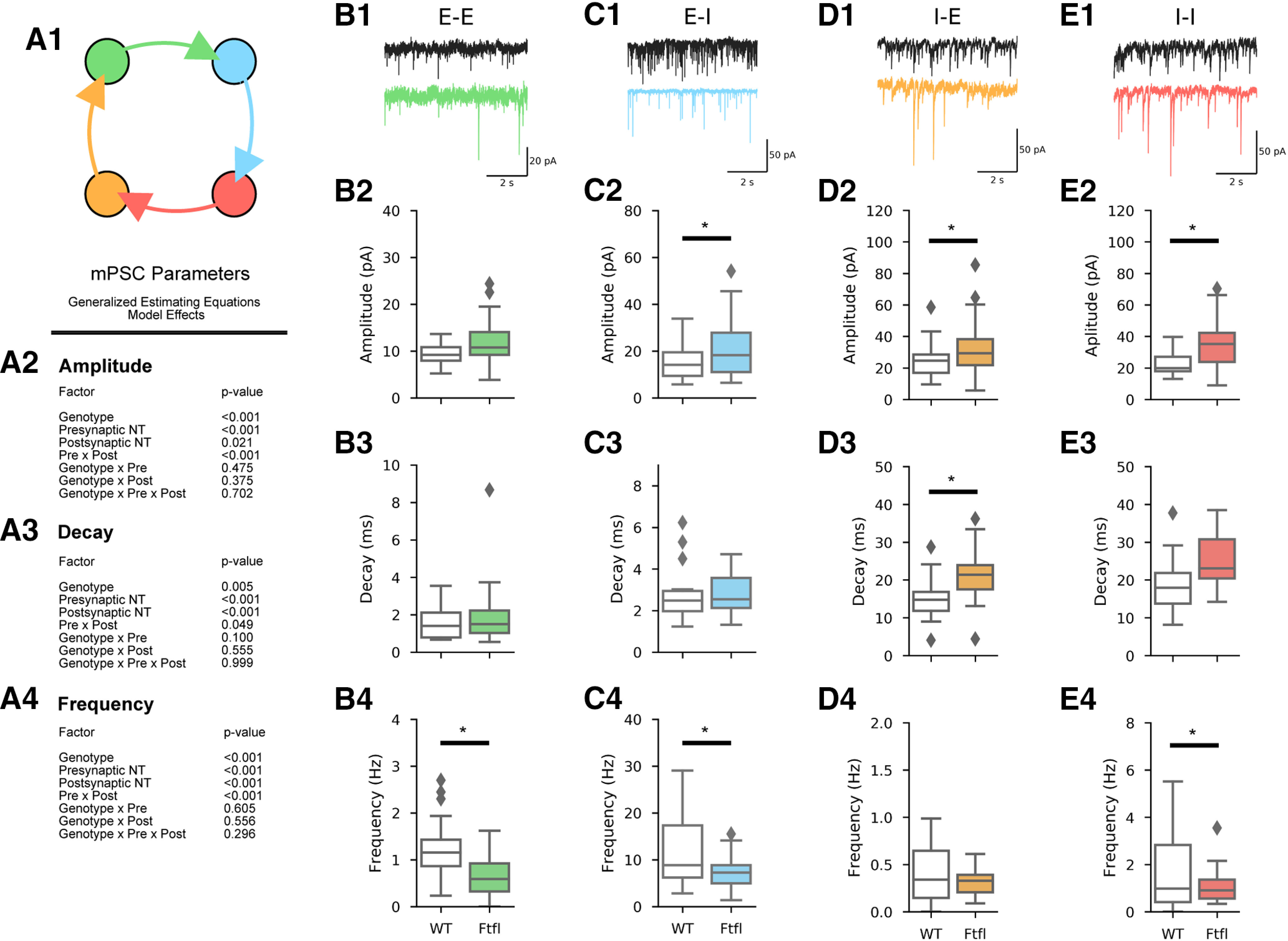
Miniature PSC frequency is reduced, and size is increased by *Dnm1*^Ftfl/Ftfl^. ***A1***, Diagram of the four motifs under study. Color scheme shown here is used throughout the paper. ***A2–A4***, Model effects from GEEs assessing each parameter of interest. ***B1***, Representative mEPSC recordings from WT (black) and Fitful (green) excitatory neurons. ***B2–B4***, Boxplots comparing amplitude, decay, and frequency of mEPSCs in WT (white, *N* = 2, *n* = 27) and Fitful (green, *N* = 2, *n* = 31) neurons. Boxes define the range, median, and IQR of the distribution. Fliers are neurons falling >1.5× of the IQR. ***C1***, Representative mEPSC recordings from WT (black) and Fitful (blue) inhibitory neurons. ***C2–C4***, Boxplots comparing amplitude, decay, and frequency of mEPSCs in WT (white, *N* = 2, *n* = 23) and Fitful (green, *N* = 2, *n* = 23) neurons. Boxes define the range, median, and IQR of the distribution. Fliers are neurons falling >1.5× of the IQR. ***D1***, Representative mIPSC recordings from WT (black) and Fitful (orange) excitatory neurons. ***D2–D4***, Boxplots comparing amplitude, decay, and frequency of mIPSCs in WT (white, *N* = 2, *n* = 27) and Fitful (orange, *N* = 2, *n* = 29) neurons. Boxes define the range, median, and IQR of the distribution. Fliers are neurons falling >1.5× of the IQR. ***E1***, Representative mIPSC recordings from WT (black, *N* = 2, *n* = 23) and Fitful (red, *N* = 2, *n* = 24) inhibitory neurons. ***E2–E4***, Boxplots comparing amplitude, charge, decay, and frequency of mIPSCs in WT (white) and Fitful (red) neurons. Boxes define the range, median, and IQR of the distribution. Fliers are neurons falling >1.5× of the IQR; *pairwise comparison *p* < 0.05. For complete pairwise comparisons data, see Extended Data Figure 1-1.

To study the synaptic effects of *Dnm1^Ftfl^*, we made cortical cultures from P1 *Dnm1^Ftfl/Ftfl^* mice and WT littermate controls. Cultures were then allowed to develop until DIV11, at which point we performed voltage clamp recordings to assess synaptic properties. Because the cortex is predominantly composed of excitatory (glutamatergic) and inhibitory (GABAergic) neurons, which give rise to four main fast synaptic motifs: E-E, I-I, and mixed motifs E-I and I-E (Fig. 1*A1*), we tested the effects of *Dnm1^Ftfl^* on each of these motifs.

10.1523/ENEURO.0269-20.2020.f1-1 Extended Data Figure 1-1 mPSC pairwise comparisons Download Figure 1-1, DOCX file.

For statistical analysis of the effect of *Dnm1^Ftfl^* on these four motifs, we used GEEs (see Materials and Methods). Presynaptic neurotransmitter, postsynaptic neuron type, and genotype were the factors, and a full-factorial analysis was run to assess how these factors impact the parameters independently, as well as assess whether genotype interacts with presynaptic or postsynaptic neuron type to produce its effects. For example, if the *Dnm1^Ftfl^* variant affected all synapses similarly, we would expect to see a statistically significant main effect of genotype with no significant interactions involving genotype. Alternatively, if the *Dnm1^Ftfl^* variant were to exclusively affect either excitatory or inhibitory synapses, we would expect to observe a statistically significant interaction between genotype and presynaptic neurotransmitter. Similarly, if the *Dnm1^Ftfl^* variant impacted only one motif, we would expect a statistically significant three-way interaction between presynaptic neurotransmitter, postsynaptic neuron type, and genotype. In each of these interaction cases, *post hoc* pairwise comparisons between motifs would then reveal which neurotransmitter or motif was affected.

First, we analyzed mPSCs by voltage clamping either excitatory or inhibitory neurons at −70 mV and applying TTX and Bic to isolate mEPSCs (Fig. 1*B1*,*C1*), or TTX and either Kyn or NBQX to isolate mIPSCs (Fig. 1*D1*,*E1*). mPSC amplitude was significantly increased in *Dnm1^Ftfl/Ftfl^* neurons, as evidenced by a significant main effect of genotype in the GEE (Fig. 1*A2*), as well as significant pairwise comparisons at each motif, except E-E (Fig. 1*B2*,*C2*,*D2*,*E2*; Extended Data Fig. 1-1). Additionally, mPSC decay time was longer for *Dnm1^Ftfl/Ftfl^* synapses at all four motifs as evidenced by a significant model effect of genotype and no interactions (Fig. 1*A*3). Only the I-E motif reached the pairwise significance threshold (Fig. 1*D3*; Extended Data Fig. 1-1). Finally, mPSC frequency was reduced in *Dnm1^Ftfl/Ftfl^* cultures, as supported by a significant effect of genotype with no interactions, and significant pairwise comparisons at three of the four motifs (Fig. 1*A4*; Extended Data Fig. 1-1). These results are similar to findings in *Dnm1/3* DKO neurons (Lou et al., 2012), and consistent with previous reports of reduced SV number but increased SV size in *Dnm1^Ftfl/Ftfl^* brains (Dhindsa et al., 2015). Taken together, these data suggest the impact of the *Dnm1^Ftfl^* variant is similar to both *Dnm1* KO and *Dnm1/3* DKO, providing support for the notion that *Dnm1^Ftfl^* is a loss-of-function variant that interferes with the function of other dynamin isoforms.

### Reduced synapse number accounts for reduced mPSC frequency

**Figure 2.**
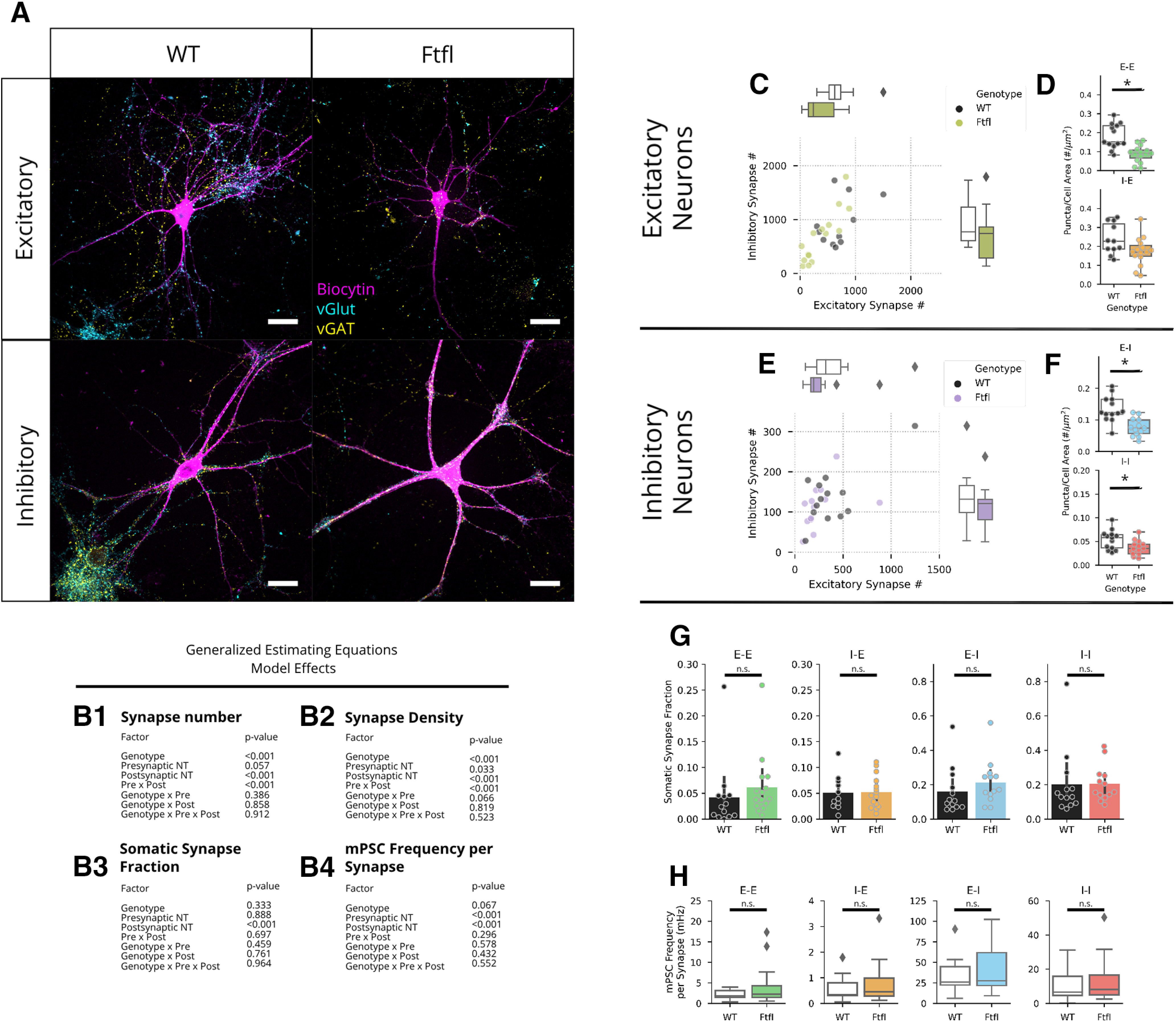
Synapse number is reduced by *Dnm1*^Ftfl/Ftfl^, and accounts for reduced mPSC frequency. ***A***, Images of biocytin-filled neurons (magenta) immunostained for vGlut1 (cyan), and vGAT (yellow). The top row shows representative excitatory neurons, and the bottom row shows representative inhibitory neurons. The left column shows representative WT neurons, and the right column shows representative Fitful neurons. Scale bars: 20 μm in length. ***B1–B4***, Model effects tested using GEEs. ***C***, Scatterplot showing excitatory synapse count (*x*-axis) and inhibitory synapse count (*y*-axis) onto excitatory neurons. Each dot represents an individual WT (black, *N* = 2, *n* = 11) or Fitful (chartreuse, *N* = 2, *n* = 15) neuron. Marginal boxplots define the median, IQR, and range along their respective axes. ***D***, Plots showing synapse density (synapses per unit area) for the E-E (top) and I-E (bottom) motifs. WT swarm and box plots are in black and white, while Fitful swarm and box plots are colored for their respective motifs. Boxplots define the median, IQR, and range. ***E***, Scatterplot showing excitatory synapse count (*x*-axis) and inhibitory synapse count (*y*-axis) onto inhibitory neurons. Each dot represents an individual WT (black, *N* = 2, *n* = 12) or Fitful (purple, *N* = 2, *n* = 13) neuron. Marginal boxplots define the median, IQR, and range along their respective axes. ***F***, Plots showing synapse density (synapses per unit area) for the E-E (top) and I-E (bottom) motifs. WT swarm and box plots are in black and white, while Fitful swarm and box plots are colored for their respective motifs. Boxplots define the median, IQR, and range. ***G***, Barplots showing the fraction of synapses located on the soma. Bars end at the mean, and error bars show the 95% confidence interval derived from bootstrap resampling. Dots represent individual neurons. ***H***, Plots showing mPSC frequency normalized to synapse number for WT (white) and Fitful (colored) neurons; *pairwise comparison *p* < 0.05. For complete pairwise comparisons data, see Extended Data Figure 2-1.

Previous research suggests that *Dnm1* KO or *Dnm1/3* DKO reduces SV number. However, other reports suggest that *Dnm1/3* DKO may alter the process of synapse formation. To investigate this in *Dnm1^Ftfl/Ftfl^* neurons, we immunostained a subset of recorded neurons filled with biocytin with antibodies against the SV-associated vesicular glutamate and GABA transporter proteins (VGLUT1 and VGAT respectively; Fig. 2*A*). This allowed us to approximate the number of excitatory and inhibitory synapses (i.e., synapses with a detectable pool of SVs) made onto excitatory and inhibitory neurons. The overall number of synapses was reduced in *Dnm1^Ftfl/Ftfl^* cultures compared with WT, as evident from the significant main effect of genotype (Fig. 2*B1*), although this did not reach significance in any of the pairwise comparisons (Fig. 2*C*,*E*; Extended Data Fig. 2-1). Normalizing the number of synapses to neuronal area similarly resulted in a significant reduction (Fig. 2*B2*), with significant pairwise comparisons at the E-E (Fig. 2*D*, top; Extended Data Fig. 2-1), E-I, and I-I motifs (Fig. 2*F*; Extended Data Fig. 2-1). There was no difference in overall neuronal area (data not shown), and no difference in the fraction of perisomatic synapses (Fig. 2*B3*,*G*; Extended Data Fig. 2-1). Finally, we calculated the frequency of mPSCs per synapse for each recorded neuron. There were no significant differences between genotypes in the mean mPSC frequency per synapse across any of the four motifs (Fig. 2*H*,*B4*; Extended Data Fig. 2-1), suggesting that the reduction in mPSC frequency was largely because of reduced synapse number.

10.1523/ENEURO.0269-20.2020.f2-1 Extended Data Figure 2-1 Synapse counts, density, and mPSC frequency Download Figure 2-1, DOCX file.

### The I-E motif is specifically affected by *Dnm1^Ftfl^*

**Figure 3.**
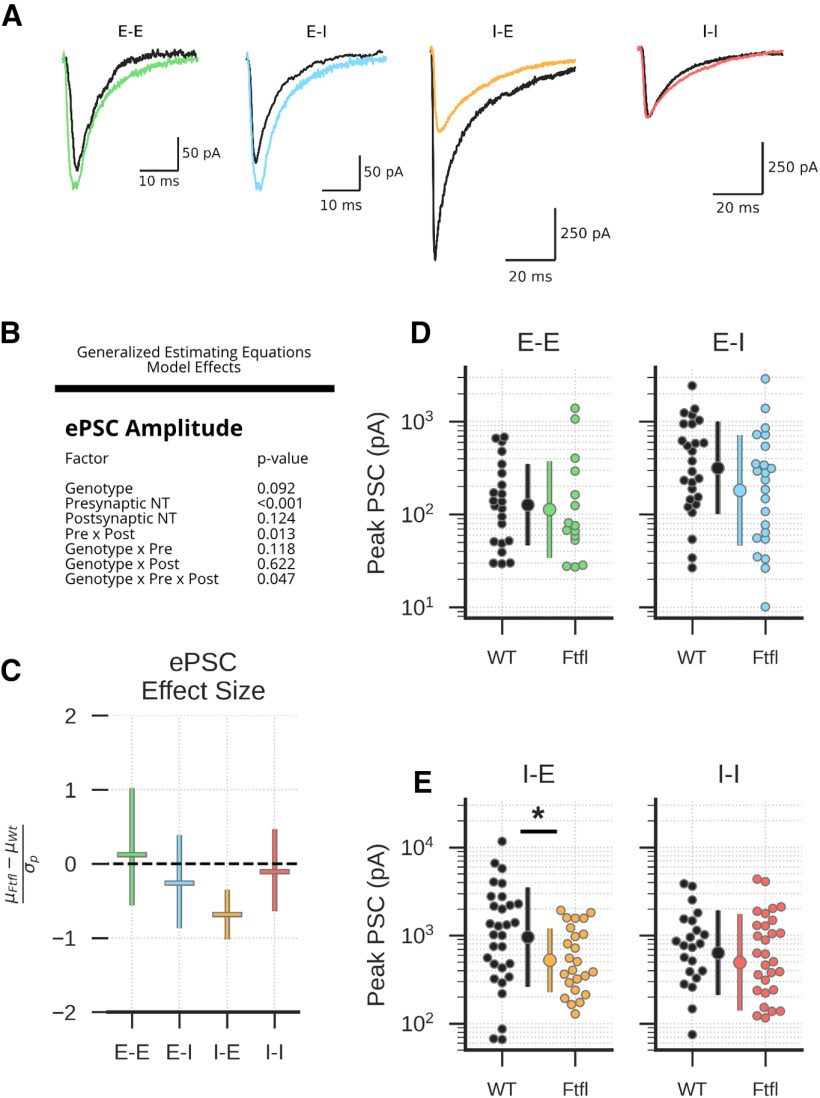
*Dnm1*^Ftfl/Ftfl^ reduces ePSC amplitude at the I-E motif. ***A***, Example ePSCs. ***B***, Model effects assessed using GEEs. ***C***, Effect sizes at each of the four motifs. Central marker and error bars represent mean effect size ± 95% CI calculated using hierarchical bootstrap resampling. ***D***, Average evoked excitatory PSCs. Swarm plots show individual synapses, and central dots and error bars represent mean ± SD (E-E: *N* = 7, WT *n* = 22, Ftfl *n* = 15; E-I: *N* = 6, WT *n* = 26, Ftfl *n* = 23). ***E***, Average evoked inhibitory PSCs. Swarm plots show individual synapses, and central dots and error bars represent mean ± SD (I-E: *N* = 7, WT *n* = 30, Ftfl *n* = 24; I-I: *N* = 5, WT *n* = 22, Ftfl *n* = 29); *pairwise comparison *p* < 0.05. For complete pairwise comparisons data, see Extended Data Figure 3-1.

Work from *Dnm1* KO neurons suggests inhibitory synapses are more vulnerable to *Dnm1* loss (Ferguson et al., 2007; Hayashi et al., 2008), and work from *Dnm1^Ftfl^* mice suggests dysfunctional inhibitory transmission is likely responsible for seizure generation (Boumil et al., 2010; Asinof et al., 2015). However, we did not detect substantial differences in the effect of *Dnm1^Ftfl/Ftfl^* on excitatory and inhibitory neurotransmission in our miniature PSC analysis, suggesting that spontaneous release of glutamatergic and GABAergic SVs is similarly affected by *Dnm1^Ftfl/Ftfl^*. To determine how evoked inhibitory synaptic transmission is particularly affected, we next performed simultaneous recordings from nearby excitatory and inhibitory neurons (Fig. 3*A*), evoked APs in each of the two neurons, and recorded the peak amplitude of the responses in the partner. Evoked post synaptic current ePSC amplitudes were unaffected at the E-E, E-I, and I-I motifs. However, eIPSC amplitude at the I-E motif was selectively reduced, as evidenced by a significant three-way interaction between genotype, presynaptic neuron type, and postsynaptic neuron type (Fig. 3*B*), as well as a significant pairwise comparison between genotypes exclusively at the I-E motif (Fig. 3*C*,*E*; Extended Data Fig. 3-1).

10.1523/ENEURO.0269-20.2020.f3-1 Extended Data Figure 3-1 ePSC pairwise comparisons Download Figure 3-1, DOCX file.

**Figure 4.**
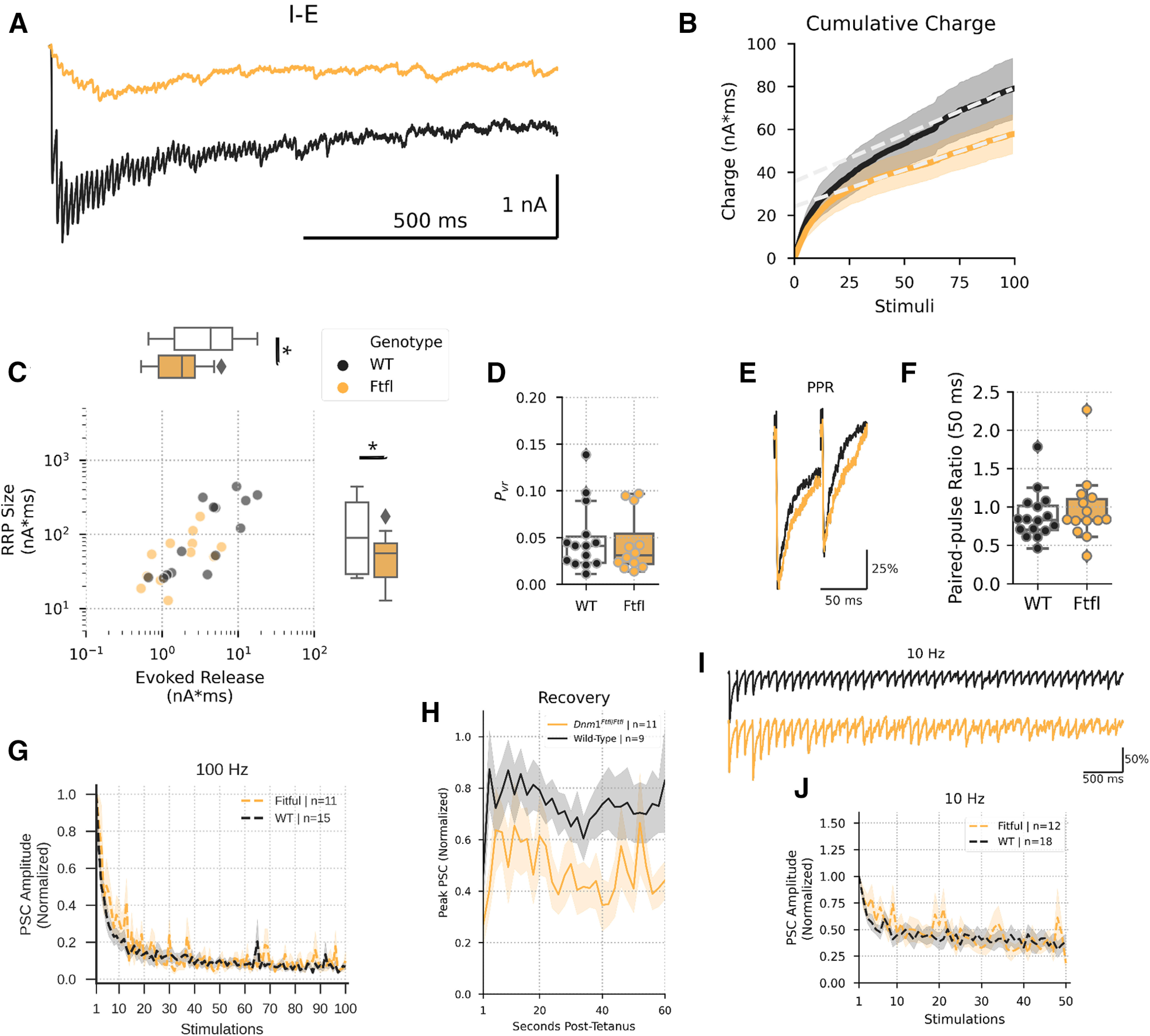
Reduced eIPSC size at the I-E motif is because of reduced RRP size, and recovery from stimulation is impaired. ***A***, Representative tetanic stimulation protocols from WT (black) and Fitful (orange) synapses. ***B***, Example cumulative charge plot; *x*-axis shows stimulus number, and *y*-axis shows the cumulative charge evoked by that stimulus. Black line and shaded region represent mean ± SEM cumulative charge for WT synapses, and orange line and shaded region represents mean ± SEM cumulative charge for Ftfl synapses. Gray dashed lines are example fits of steady-state synaptic release during tetanus used to calculate RRP size. ***C***, Scatterplot showing charge contained in the first stimulus (*x*-axis) and charge contained in the RRP (*y*-axis) of WT (black, *N* = 5, *n* = 14) and Fitful (orange, *N* = 5, *n* = 12) synapses. Marginal boxplots show median and IQR of their respective axes. Fliers are synapses that exceed 1.5× the IQR. ***D***, Release probability at WT (black) and Fitful (orange) synapses. Dots represent individual synapses, and boxplots show range, median, and IQR. Fliers are synapses that exceed 1.5× the IQR. ***E***, Example paired-pulse ratio IPSCs evoked with a 50-ms delay. Traces are normalized to the peak of the first IPSC. ***F***, Box and swarm plots showing 50-ms paired-pulse ratios. Dots represent individual WT (black) and Fitful (orange) synapses, and boxplots show range, median, and IQR. ***G***, Peak amplitudes of synaptic responses during 100-Hz stimulation for WT (black) and Fitful (orange) synapses, normalized to the peak of the first response. Lines and shaded regions represent mean ± SEM of responses. ***H***, Timeline of synaptic recovery from 100-Hz stimulation for WT (black) and FItful (orange) synapses. Peak amplitudes of each response were normalized to the baseline response of the given neuron. Lines and shaded regions represent mean ± SEM of responses. ***I***, Example IPSCs evoked by 10-Hz stimulation. Traces are normalized to the peak of the first IPSC. ***J***, Peak amplitudes of synaptic responses during 10-Hz stimulation for WT (black) and Fitful (orange) synapses, normalized to the peak of the first response. Lines and shaded regions represent mean ± SEM of responses; *pairwise comparison *p* < 0.05. For complete pairwise comparisons data, see Extended Data Figures 4-1, 4-2.

To understand the mechanism of dysfunction at the I-E motif, we next quantified the size of the RRP and the probability of vesicle release (P_vr_) using high-frequency stimulation (HFS; Kaeser and Regehr, 2017). Briefly, inhibitory neurons were stimulated at 100 Hz (Fig. 4*A*), and the cumulative evoked synaptic charge onto the paired excitatory neurons was plotted across stimuli to assess the SV priming rate (Fig. 4*B*), which then allowed us to estimate the size of the RRP and the P_vr_. As expected, we found that the evoked charge of the first IPSC in the train was significantly smaller in *Dnm1^Ftfl/Ftfl^* pairs (Fig. 4*C*; Extended Data Fig. 4-1). The RRP was also significantly reduced (Fig. 4*C*; Extended Data Fig. 4-1); however, the P_vr_ was unaffected (Fig. 4*D*; Extended Data Fig. 4-1), as was the paired-pulse ratio (Fig. 4*E*,*F*; Extended Data Fig. 4-2). This suggests that the reduced eIPSC amplitude observed at the I-E motif is because of reduced SV RRP size, not changes in the kinetics of SV release.

10.1523/ENEURO.0269-20.2020.f4-1 Extended Data Figure 4-1 RRP parameters pairwise comparisons Download Figure 4-1, DOCX file.

10.1523/ENEURO.0269-20.2020.f4-2 Extended Data Figure 4-2 Synaptic dynamics pairwise comparisons Download Figure 4-2, DOCX file.

Because previous work suggested that *Dnm1* disruption altered short-term plasticity and slowed recovery from repetitive stimulation, we next assessed the time course of depression and recovery of eIPSCs onto excitatory neurons. *Dnm1^Ftfl/Ftfl^* synapses did not depress to a greater extent than WT synapses during either 10 Hz (5 s; Fig. 4*I*,*J*; Extended Data Fig. 4-2) or 100 Hz (1 s) stimulation (Fig. 4*G*; Extended Data Fig. 4-2). They did, however, demonstrate significantly slower recovery of the eIPSC amplitude following 100-Hz stimulation (Fig. 4*E*; Extended Data Fig. 4-2), consistent with the proposed role for *Dnm1* in SV endocytosis following HFS (Ferguson et al., 2007; Armbruster et al., 2013). Taken together, these results suggest the *Dnm1^Ftfl^* variant has motif-specific effects that disproportionately impacts inhibition. In particular, maintenance of the RRP at the I-E motif is impaired, leading to reduced inhibitory restraint of excitatory neurons.

### *Dnm1^Ftfl/Ftfl^* networks are not hyperactive

**Figure 5.**
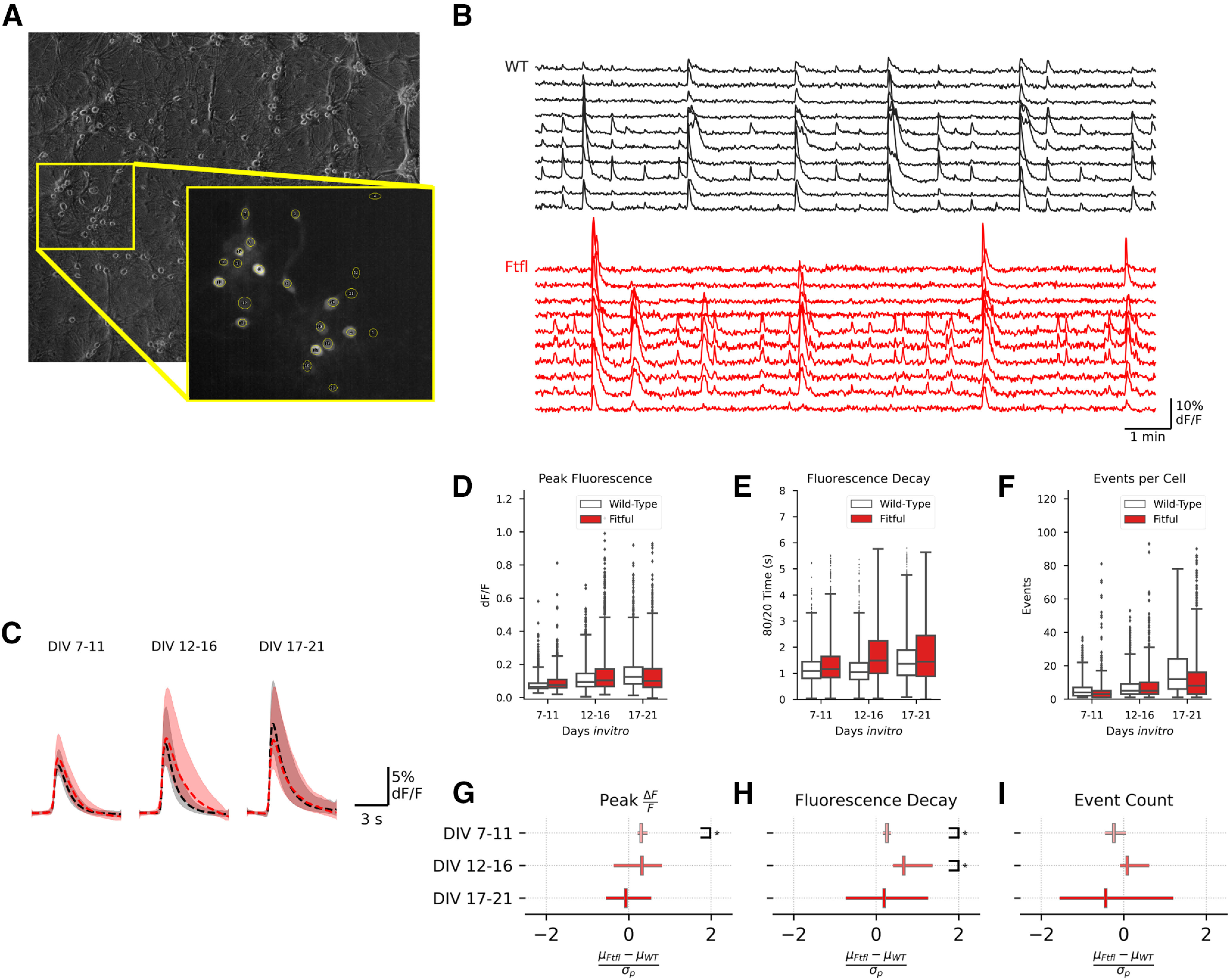
Calcium Imaging shows little evidence of increased excitability. ***A***, Example imaging field. Inset, Maximum fluorescence projection of the outlined region showing manually drawn ROIs. ***B***, Example fluorescence traces from WT (black) and Fitful (red) neurons at DIV13. ***C***, Average fluorescence events for Fitful (red, *N* = 3, *n* = 6788) and WT (black, *N* = 3, *n* = 8850) neurons across developmental epochs. Lines represent median fluorescence across all events, and the shaded region defines the IQR. ***D***, Peak fluorescence boxplots across developmental epochs for WT (white) and Fitful (red) neurons. Boxplots define the range, median, and IQR for all observed neurons. Fliers represent neurons falling outside 3× of the IQR. ***E***, Boxplots displaying the 80/20 fluorescence decay time for each cell in WT (white) and Fitful (red) cultures. Boxplots define the range, median, and IQR for all observed neurons. Fliers represent neurons falling outside 3× of the IQR. ***F***, Boxplots displaying the number of events observed for each cell in WT (white) and Fitful (red) cultures. Boxplots define the range, median, and IQR for all observed neurons. Fliers represent neurons falling outside 3× of the IQR. ***G–I***, Plots showing the effect size of the Fitful variant at each of the developmental epochs on peak change in flourescence (***G***), fluorescence decay (***H***), and events per cell (***I***). Central marker and error bars represent mean effect size ± 95% CI calculated using hierarchical bootstrap resampling; *pairwise comparison *p* < 0.05. For complete model effects and pairwise comparisons data, see Extended Data Figures 5-1, 5-2.

Because impairment of the I-E motif suggested inhibitory transmission was compromised, we wanted to assess the level of activity in cultured neural networks harboring the *Dnm1^Ftfl^* variant. Some of the *Dnm1^Ftfl/Ftfl^* synaptic alterations, such as reduction in the number of E-E synapses, would be predicted to decrease network activity, while others, such as the reduction of synaptic strength at the I-E motif, may increase network activity. To determine the effect of *Dnm1^Ftfl/Ftfl^* on activity levels in the cultured network, we expressed GCaMP6f in excitatory neurons and imaged their fluorescence daily for 20 min from DIV7 through DIV21. For analysis purposes, we divided the imaging timeline into early (DIV7–DIV11), middle (DIV12–DIV16), and late (DIV17–DIV21) developmental epochs. Following imaging, ROIs were manually drawn around neurons in ImageJ (Fig. 5*A*) to extract fluorescence traces (Fig. 5*B*). Fluorescence events that likely reflected AP firing were then detected with Python using a derivative threshold.

10.1523/ENEURO.0269-20.2020.f5-1 Extended Data Figure 5-1 Calcium imaging model effects Download Figure 5-1, DOCX file.

We first compared the event amplitudes for individual neurons in Dnm1^Ftfl/Ftfl^ and WT networks. Comparisons of epochs showed that the peak amplitude of fluorescent events increased as cultures developed in both the WT and *Dnm1^Ftfl/Ftfl^* groups (Fig. 5*C*,*D*,*G*; Extended Data Fig. 5-1). Although there was not a significant effect of genotype on event amplitude (Extended Data Fig. 5-1), comparisons between genotypes at each epoch found a significant increase in event amplitude in the first epoch in *Dnm1^Ftfl/Ftfl^* neurons (Fig. 5*C*,*D*,*G*; Extended Data Fig. 5-2). We also measured the 80–20% fluorescence decay time. We found a significant interaction between genotype and developmental epoch. Pairwise comparisons showed that the decay time was significantly longer in *Dnm1^Ftfl/Ftfl^* neurons in the first two developmental epochs (Fig. 5*E*,*H*; Extended Data Fig. 5-1). Taken together, these data suggest there may be (1) a small increase in the number of APs fired per event, (2) altered timing of APs within events, or (3) altered calcium dynamics in *Dnm1^Ftfl/Ftfl^* neurons, possibly because of weakened inhibition of excitatory neurons.

10.1523/ENEURO.0269-20.2020.f5-2 Extended Data Figure 5-2 Calcium imaging pairwise comparisons Download Figure 5-2, DOCX file.

We next measured the number of events that occurred per neuron in each imaging session. This event rate also increased in both groups as the cultures developed, but there was not a significant effect of genotype overall (Fig. 5*F*,*I*; Extended Data Fig. 5-1). We did observe a significant genotype by epoch interaction (Extended Data Fig. 5-1), but pairwise comparisons at each individual epoch were not statistically significant, and mean differences were very small. Taken together, this suggests *Dnm1^Ftfl/Ftfl^* and WT cultures have similar developmental trajectories and similar levels of overall activity.

### Spontaneous PSC frequency is reduced in *Dnm1^Ftfl/Ftfl^* cultures

**Figure 6.**
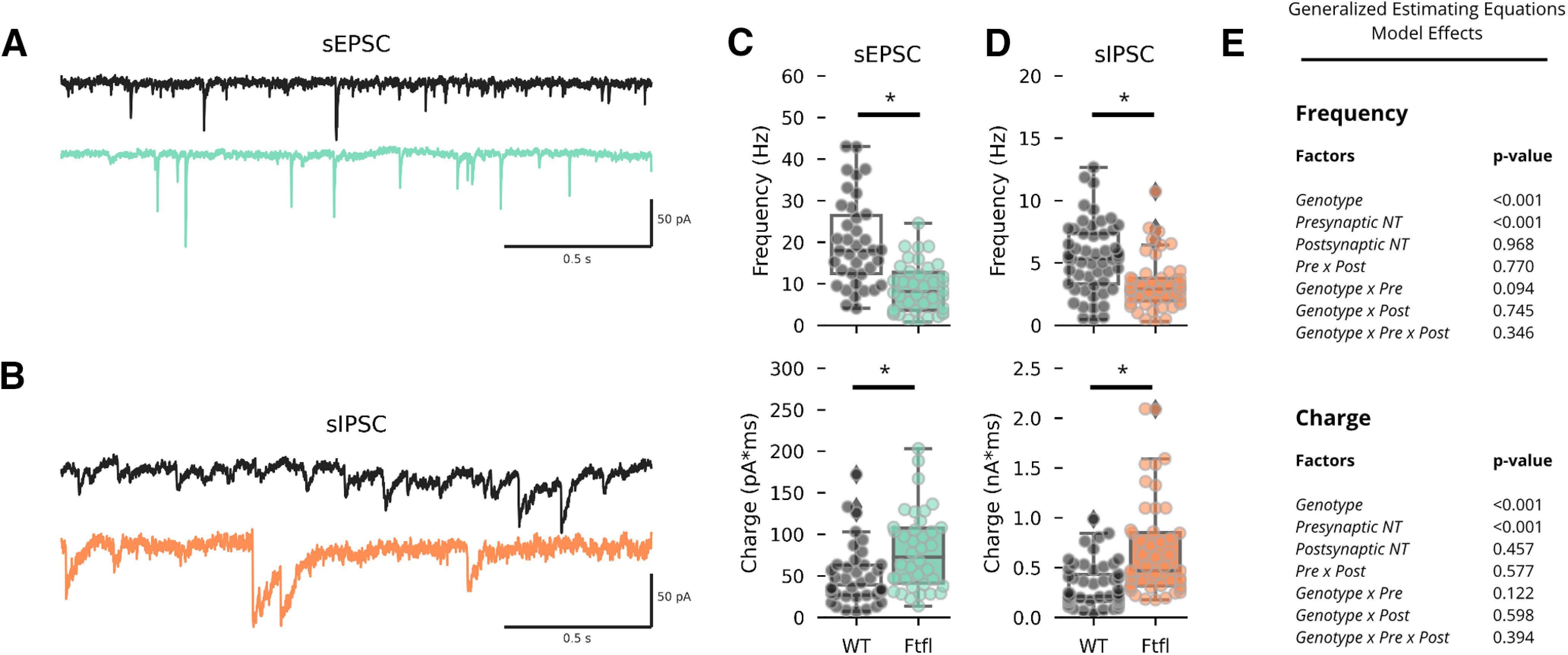
Spontaneous PSCs are larger and less frequent in *Dnm1*^Ftfl/Ftfl^ cultures. ***A***, Representative voltage-clamp recordings of sEPSCs from WT (black) and Fitful (teal) neurons. ***B***, Representative voltage-clamp recordings of sIPSCs from WT (black) and Fitful (orange) neurons. ***C***, Box and swarm plots showing sEPSC frequency (top) and sEPSC charge (bottom) for WT (black, *N* = 3, *n* = 38) and Fitful (teal, *N* = 3, *n* = 38) neurons. Dots represent individual neurons. Boxplots show range, median, and IQR, with fliers representing neurons beyond 1.5× the IQR. ***D***, Box and swarm plots showing sIPSC frequency (top) and sIPSC charge (bottom) for WT (black, *N* = 3, *n* = 53) and Fitful (orange, *N* = 3, *n* = 47) neurons. Dots represent individual neurons. Boxplots show range, median, and IQR, with fliers representing neurons beyond 1.5× the IQR. ***E***, Model effects tested using GEEs; *pairwise comparison *p* < 0.05. For complete pairwise comparisons data, see Extended Data Figure 6-1.

To further assess differences in activity electrophysiologically, we recorded excitatory and inhibitory spontaneous PSCs (sEPSCs and sIPSCs). Briefly, we voltage clamped neurons between DIV13–DIV16 at −70 mV and synaptic events were recorded in Bic to isolate sEPSCs (Fig. 6*A*) or Kyn to isolate sIPSCs (Fig. 6*B*). sPSC frequency was significantly reduced by the *Dnm1^Ftfl/Ftfl^* variant, as evidenced by a significant main effect of genotype. Pairwise comparisons between genotypes revealed significantly reduced sPSC frequency across all motifs at *Dnm1^Ftfl/Ftfl^* synapses (Fig. 6*C*,*D*, top, *E*; Extended Data Fig. 6-1), supporting the idea that neural activity is not increased, and possibly reduced, in *Dnm1^Ftfl/Ftfl^*cultures. Also, in agreement with the mPSC results, sPSC charge was significantly increased across all motifs at *Dnm1^Ftfl/Ftfl^* synapses (Fig. 6*C*,*D*, bottom, *E*; Extended Data Fig. 6-1).

10.1523/ENEURO.0269-20.2020.f6-1 Extended Data Figure 6-1 sPSC pairwise comparisons Download Figure 6-1, DOCX file.

### The rate and timing of AP firing is altered in *Dnm1^Ftfl/Ftfl^* neurons

**Figure 7.**
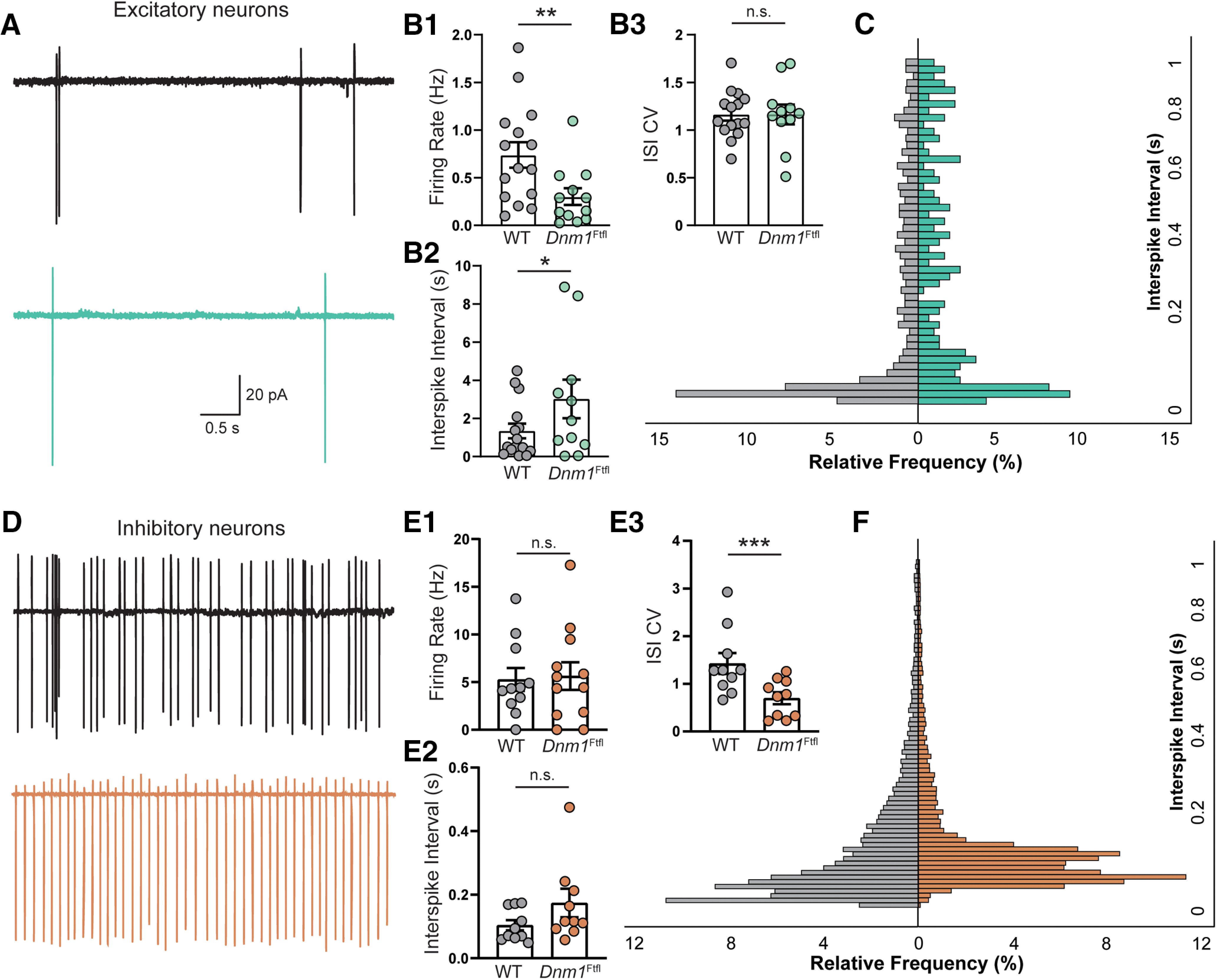
*Dnm1*^Ftfl/Ftfl^ alters the frequency and timing of spontaneous APs. ***A***, Example traces of the current response during cell-attached voltage clamp recordings of excitatory neurons from WT (black) and *Dnm1*^Ftfl^ (green) cultures. ***B1***, Plot of the mean firing rate (MFR) of WT and *Dnm1*^Ftfl^ excitatory neurons. ***B2***, Plot of the median ISI from each group. ***B3***, Plot of the coefficient of variation (CV) of the ISI. ***C***, A relative frequency histogram of all ISIs (log scale) collected from WT (left) and *Dnm1*^Ftfl^ (right) excitatory neurons. ***D***, Example traces of the current response during cell-attached voltage clamp recordings of inhibitory neurons from WT (black) and *Dnm1*^Ftfl^ (orange) cultures. ***E1***, Plot of the MFR of WT and *Dnm1*^Ftfl^ excitatory neurons. ***E2***, Plot of the median ISI from each group. ***E3***, Plot of the CV of the ISI. ***F***, Relative frequency histogram of all ISIs (log scale) collected from WT (left) and *Dnm1*^Ftfl^ (right) inhibitory neurons. For all plots, each dot represents the mean or median value from one neuron; *pairwise comparison *p* < 0.05; **pairwise comparison *p* < 0.01, ***pairwise comparison *p* < 0.001. For complete pairwise comparisons data, see Extended Data Figure 7-1.

Although sPSC frequency is reduced (Fig. 6), these events are a combination of both mPSCs and AP-driven PSCs, making it unclear whether decreased neural activity contributes to the reduction in sPSCs. Also, Ca^2+^ transients were altered in *Dnm1^Ftfl/Ftfl^* neurons (Fig. 5), suggesting potential differences in AP timing. We therefore performed cell-attached recordings of excitatory or inhibitory neurons between DIV12 and DIV14 to detect spontaneous APs and examine whether their rate and timing were altered (Fig. 7).

10.1523/ENEURO.0269-20.2020.f7-1 Extended Data Figure 7-1 Cell attached pairwise comparisons Download Figure 7-1, DOCX file.

We found that *Dnm1^Ftfl/Ftfl^* excitatory neurons fired fewer spontaneous APs than WT controls (Fig. 7*A*; Extended Data Fig. 7-1), as measured by a reduction in the mean firing rate (Fig. 7*B1*; Extended Data Fig. 7-1) and an increase in median interspike interval (ISI; Fig. 7*B2*; Extended Data Fig. 7-1), further suggesting an overall reduction in activity in *Dnm1^Ftfl/Ftfl^*cultures. To compare the timing of APs, we plotted a histogram of the relative frequencies of ISIs (Fig. 7*C*), which had significantly different distributions (Kolmogorov–Smirnov test, *p* < 0.0001, D = 0.185). Visual inspection of the histograms showed relatively fewer APs with short ISIs (0–60 ms) in *Dnm1^Ftfl/Ftfl^* neurons, but relatively more APs at 100- to 160-ms ISIs, suggesting that the increase in decay time of the Ca^2+^ transients may be because of greater spacing of APs within an event. The coefficient of variation of the ISI was not different between genotypes, indicating no change in the regularity of APs (Fig. 7*B3*; Extended Data Fig. 7-1).

The mean firing rate of *Dnm1^Ftfl/Ftfl^* inhibitory neurons was not different from WT neurons (Fig. 7*D*,*E1*; Extended Data Fig. 7-1). The distribution of ISIs was, however, altered (Kolmogorov–Smirnov test, *p* < 0.0001, D = 0.234), with a shift in the relative frequency of short latency ISIs (< 200 ms) to longer values (Fig. 7*F*) in *Dnm1^Ftfl/Ftfl^* inhibitory neurons. The coefficient of variation of the ISI was also different between genotypes, suggesting an increased regularity in the AP firing of *Dnm1^Ftfl/Ftfl^* inhibitory neurons (Fig. 7*D*,*E3*; Extended Data Fig. 7-1).

### Seizure-like events (SLEs) are less severe in *Dnm1^Ftfl/Ftfl^* cultures

**Figure 8.**
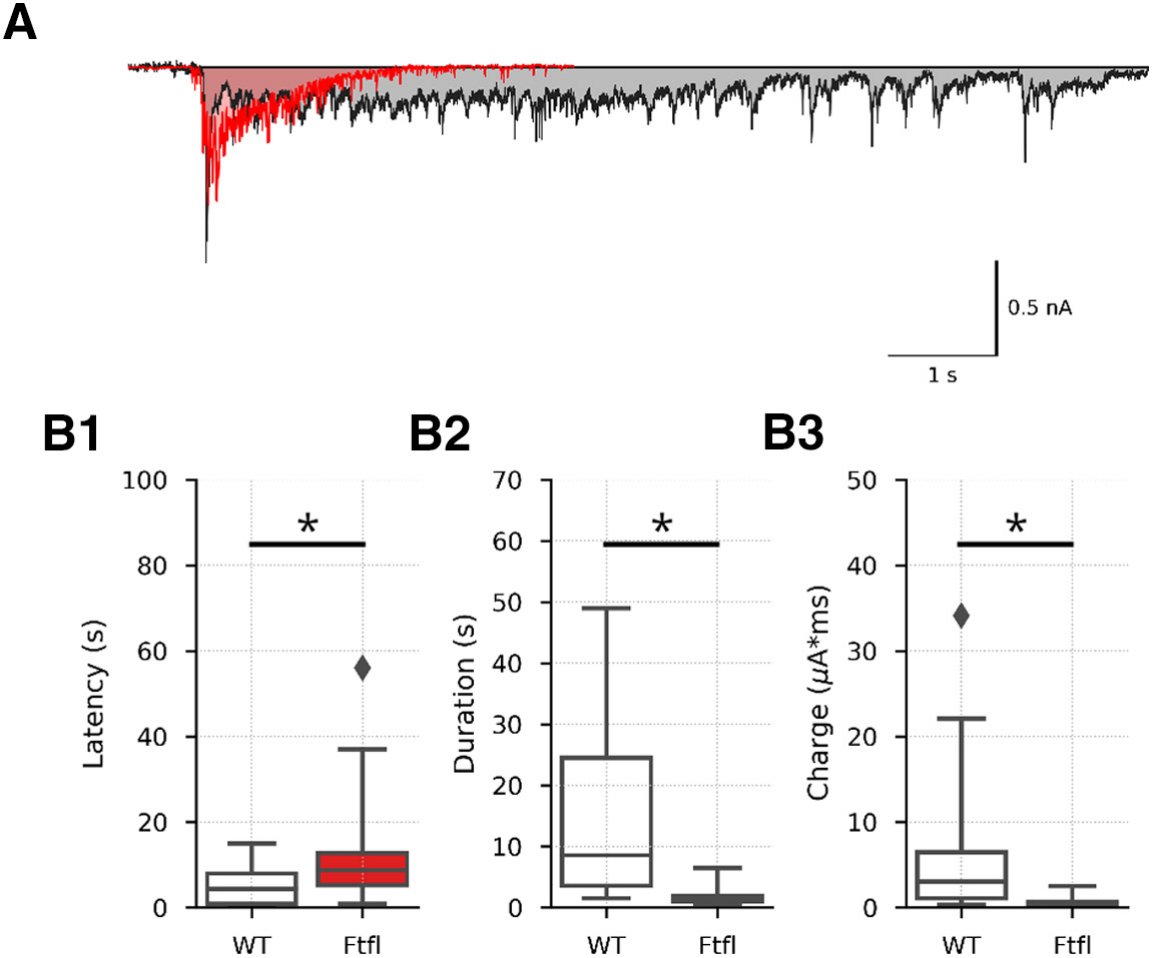
Bic-induced SLEs are shorter in *Dnm1*^Ftfl/Ftfl^ cultures. ***A***, Representative Bic-induced SLEs from WT (black, *N* = 3, *n* = 48) and Fitful (red, *N* = 3, *n* = 50) neurons aligned to SLE onset. ***B***, Plots showing latency to SLE onset. Boxplots define the range, median, and IQR of the distribution. Fliers are 1.5× the IQR. ***C***, Plots showing duration of SLEs. Boxplots define the range, median, and IQR of the distribution. ***D***, Plots showing charge evoked by SLEs. Boxplots define the range, median, and IQR of the distribution. Fliers are 1.5× the IQR; *pairwise comparison *p* < 0.05. For complete pairwise comparisons data, see Extended Data Figure 8-1.

The application of Bic to neuronal cultures often induced sustained oscillating excitatory events in voltage clamp recordings (Fig. 8*A*), which have been previously likened to seizures (Furshpan and Potter, 1989; Khalilov et al., 1997; Colombi et al., 2013). We therefore compared these events in WT and *Dnm1^Ftfl/Ftfl^* cultures to determine whether there were differences in the generation and properties of these SLEs. We found that SLEs occurred in 96% of WT and 93% of *Dnm1^Ftfl/Ftfl^* recordings, suggesting there is no difference in the propensity to have an SLE. However, the latency to SLE onset was significantly longer in *Dnm1^Ftfl/Ftfl^* cultures (Fig. 8*B1*; Extended Data Fig. 8-1). Additionally, the duration (Fig. 8*B2*; Extended Data Fig. 8-1) and charge contained in SLEs (Fig. 8*B3*; Extended Data Fig. 8-1) was significantly reduced in *Dnm1^Ftfl/Ftfl^* cultures. Together, these data suggest *Dnm1^Ftfl/Ftfl^* cultures, suggesting that Dnm1^Ftfl/Ftfl^ cultures are less capable of sustaining the hyperactive state.

10.1523/ENEURO.0269-20.2020.f8-1 Extended Data Figure 8-1 SLE pairwise comparisons Download Figure 8-1, DOCX file.

To determine whether the reduced activity occurred because of changes in intrinsic excitability of neurons, we performed current clamp recordings and injected sequentially increasing current steps to assess passive membrane properties and to induce APs. We found no differences in the resistance or capacitance of neurons, nor did we see differences in AP threshold or kinetics that would suggest neurons were hypoexcitable or hyperexcitable (Table 1). Thus, this reduction of activity and SLE duration is not attributable to altered neuronal excitability. Together, these observations suggest that, despite impairment of the I-E motif, *Dnm1^Ftfl/Ftfl^* cultures are not more active than WT controls. Further, *Dnm1^Ftfl/Ftfl^* cultures are less able to sustain SLEs.

**Table 1:** AP and passive membrane properties

	Glutamatergic neurons				GABAergic neurons			
	Control, *n* = 58	Fitful, *n* = 48	*p* value	95% CI of difference	Control, *n* = 36	Fitful, *n* = 48	*p* value	95% CI of difference
V_(rest)_ (mV)	−61.3 ± 2.06	−61.5 ± 2.08	0.871	−2.33 to 2.75	−61.5 ± 2.12	−61.9 ± 2.07	0.739	−2.1 to 2.96
AP threshold (mV)	−37.5 ± 1.0	−36.4 ± 0.99	0.215	−2.78 to 0.62	−39.2 ± 1.1	−39.3 ± 1.0	0.840	−1.61 to 1.97
AP half-width (ms)	2.5 ± 0.12	2.5 ± 0.12	0.986	−0.3 to 0.3	1.41 ± 0.07	1.44 ± 0.07	0.71	−0.13 to 0.2
AP Peak (mV from 0)	26.2 ± 1.71	23.2 ± 1.57	0.074	−6.29 to 0.29	30.84 ± 2.12	27.24 ± 1.75	0.065	−7.41 to 0.2
AHP amp. (mV)	−53.0 ± 1.11	−53.9 ± 1.16	0.424	−1.26 to 3.06	−59.2 ± 1.32	−60.6 ± 1.26	0.273	−1.05 to 3.74

	Control, *n* = 31	Fitful, *n* = 30	*p* value	95% CI of difference	Control, *n* = 40	Fitful, *n* = 52	*p* value	95% CI of difference
Resistance (MΩ)	218.2 ± 21.2	195.0 ± 19.0	0.384	−75.4 to 29.0	197.3 ± 17.6	209.1 ± 16.9	0.580	−30.0 to 53.6
Capacitance (pF)	99.76 ± 11.34	114.46 ± 12.91	0.249	−10.3 to 39.7	102.89 ± 11.34	92.43 ± 9.58	0.267	−28.93 to 8.0

Mean ± SEM for each of the measures parameters; *p* values and 95% CIs were derived from pairwise comparisons of the estimated marginal means from GEEs.

### *Dnm1^Ftfl/Ftf^*^l^ neurons deteriorate and die

One of the most prominent and consistent features we observed in *Dnm1^Ftfl/Ftfl^* cultures was the deterioration and ultimate death of neurons. Beginning around DIV14, the appearance of *Dnm1^Ftfl/Ftfl^* cultures began to change in ways that indicated neuron health was compromised. *Dnm1^Ftfl/Ftfl^* neurons began to aggregate into tight clusters and developed granular structures, presumably lipid bodies, characteristic of deteriorating cells. Ultimately, *Dnm1^Ftfl/Ftfl^* neurons would deteriorate to the point that individual neurons were difficult to morphologically resolve.

**Figure 9.**
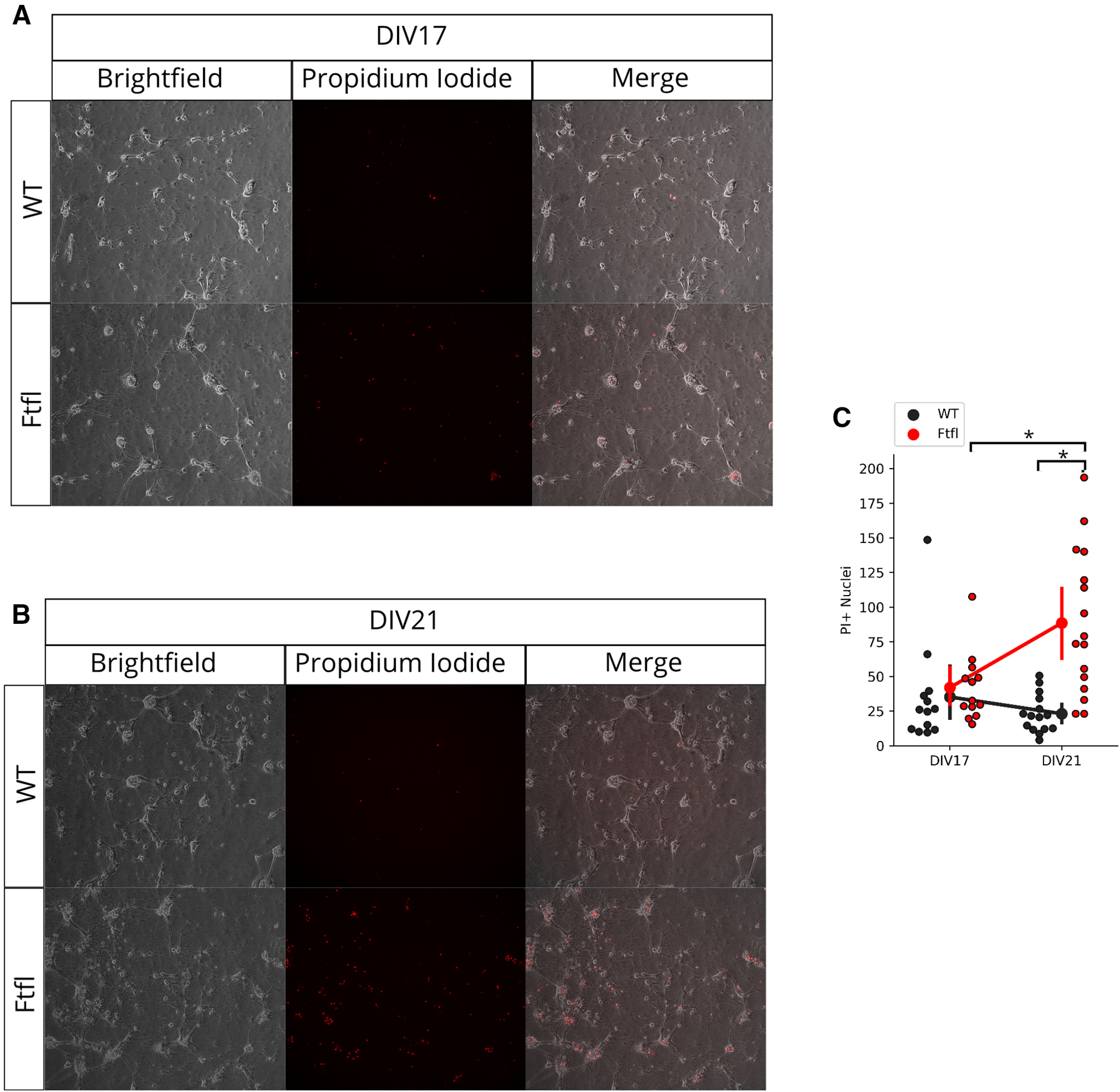
*Dnm1*^Ftfl/Ftfl^ causes neuronal degeneration. ***A***, Representative brightfield (left), PI (center), and merged (right) images for WT (top) and Fitful (bottom) cultures at DIV17. ***B***, Representative brightfield (left), PI (center), and merged (right) images for WT (top) and Fitful (bottom) cultures at DIV21. ***C***, Counts of PI-positive nuclei (average counts of two blind observers) for WT (black, DIV17: *N* = 2, *n* = 13; DIV21: *N* = 2, *n* = 15) and Fitful (red, DIV17: *N* = 2, *n* = 13; DIV21: *N* = 2, *n* = 15) images. Each dot represents the count for an individual field of view. Markers and error bars represent mean ± 95% CI calculated through bootstrap resampling; *pairwise comparison *p* < 0.05. For complete model effects and pairwise comparisons data, see Extended Data Figures 9-1, 9-2.

To quantify this phenomenon, we performed PI labeling of live cultures at DIV17 and DIV21. PI is a membrane-impermeant red fluorescent dye widely used to label dead neurons with compromised membranes (Aras et al., 2008). Unsurprisingly, we found that the number of PI+ nuclei per field of view increased in *Dnm1^Ftfl/Ftfl^* cultures from DIV17 to DIV21 (Fig. 9*A*,*B*; Extended Data Fig. 9-1). This led to a significant difference between WT and *Dnm1^Ftfl/Ftfl^* cultures at DIV 21, with *Dnm1^Ftfl/Ftfl^* cultures having far more PI+ nuclei (Fig. 9*B*,*C*; Extended Data Fig. 9-2). This observation suggests that *Dnm1^Ftfl/Ftfl^* cultures degenerate, and ultimately die, between two and three weeks *in vitro*. Interestingly, our observations parallel the timeline of death of *Dnm1^Ftfl/Ftfl^* mice, which begin to show signs of deterioration around P14 and ultimately die around P21.

10.1523/ENEURO.0269-20.2020.f9-1 Extended Data Figure 9-1 Cell death model effects Download Figure 9-1, DOCX file.

10.1523/ENEURO.0269-20.2020.f9-2 Extended Data Figure 9-2 Cell death pairwise comparisons Download Figure 9-2, DOCX file.

### *Dnm1^Ftfl^* has differential effects on inhibitory and excitatory neuron viability

**Figure 10.**
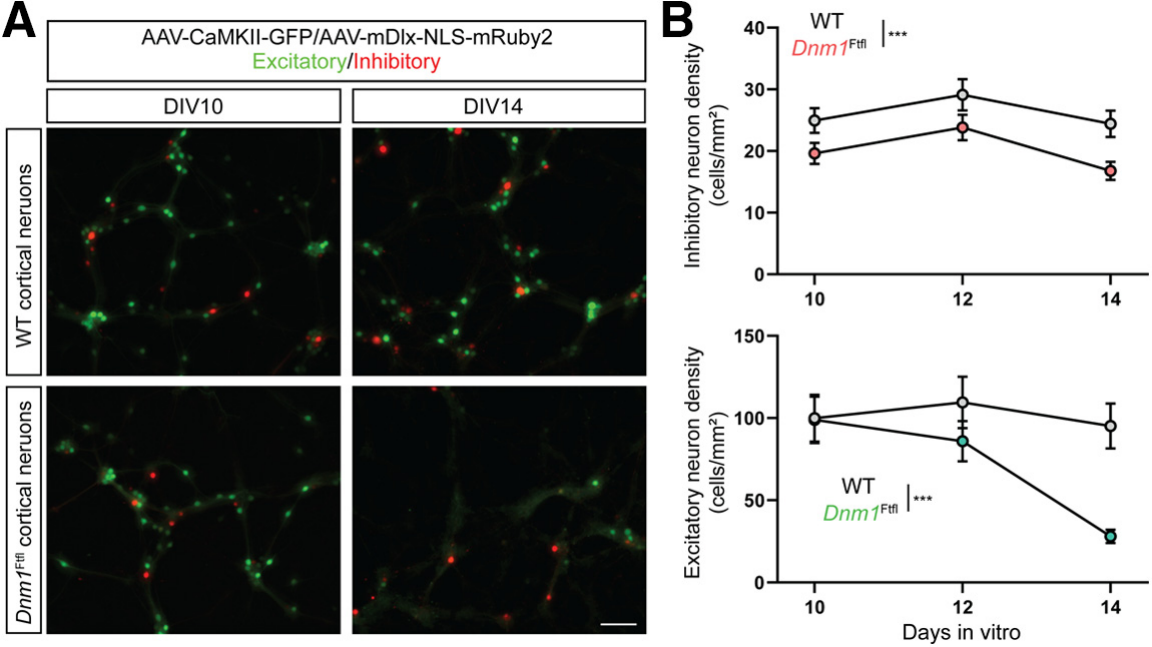
*Dnm1*^Ftfl/Ftfl^ reduces the density of inhibitory and excitatory neurons in a time-dependent manner. ***A***, Example images of WT (top) and *Dnm1*^Ftfl^ (bottom) neurons identified as excitatory via their expression of eGFP driven by the CaMKII promoter, or inhibitory via expression of mCherry driven by Dlx5/6, at DIV10 (left) and DIV14 (right). ***B***, Plots showing the density (mean ± SEM) of inhibitory (top) and excitatory (bottom) neurons as a function of days *in vitro* (DIV). Scale bar: 100 μm; ***indicates a significant effect of genotype on inhibitory neuron density and significant genotype × DIV interaction for excitatory neurons. For complete pairwise comparisons data, see Extended Data Figure 10-1.

A previous study showed that deficits in inhibitory neurons in *Dnm1*^Ftfl^ mice cause seizures and death, whereas deficits in excitatory neurons cause behavioral comorbidities, suggesting that the inhibitory neurons may be more susceptible to neuronal degeneration (Asinof et al., 2015). To test whether loss of inhibitory or excitatory neurons was occurring, we applied two AAVs to the neurons that have been shown to differentially mark excitatory and inhibitory neuron populations and then quantified the density of the two populations at DIV10, DIV12, and DIV14 (Fig. 10*A*).

10.1523/ENEURO.0269-20.2020.f10-1 Extended Data Figure 10-1 Cell counts model effects Download Figure 10-1, DOCX file.

At DIV10, inhibitory neurons comprised 19.9 ± 2.1% of all labeled neurons in WT cultures, similar to reported percentages in the mature cortex *in vivo*, while they were 16.2 ± 1.7% of labeled neurons in *Dnm1^Ftfl/Ftfl^* cultures, which was not significantly different. Across all time points, the density of inhibitory neurons was significantly lower in *Dnm1^Ftfl/Ftfl^* cultures, as there was a significant effect of genotype (Fig. 10*B*; Extended Data Fig. 10-1). The density of *Dnm1^Ftfl/Ftfl^* inhibitory neurons did not, however, decrease with time, as evidenced by no significant interaction between DIV and genotype (Fig. 10*B*; Extended Data Fig. 10-1). For excitatory neurons, the density was equal at DIV10, and only slightly lower at DIV12. By DIV14, however, excitatory neuron density was much lower in *Dnm1^Ftfl/Ftfl^* cultures, which was confirmed by a significant DIV × genotype interaction and pairwise significance at DIV14 (Fig. 10*B*; Extended Data Fig. 10-1). Overall, these results suggest *Dnm1^Ftfl/Ftfl^* may cause a small reduction in the overall numbers of inhibitory neurons, but that the widespread neuronal death we observed during week two *in vitro* is because of loss of large numbers of excitatory neurons.

## Discussion

Prior work on *Dnm1* demonstrated its role in regulating both synaptic transmission and SV recycling (Ferguson et al., 2007; Hayashi et al., 2008; Armbruster et al., 2013). More recently, human genetic studies established *DNM1* variants as causing DEEs such as infantile spasms and Lennox–Gastaut syndrome (EuroEPINOMICS-RES Consortium, Epilepsy Phenome/Genome Project, Epi4K Consortium, 2014; von Spiczak et al., 2017). These same studies also showed that synaptic transmission is the process most likely to be affected by DEE-causing gene variants, suggesting that synaptic changes are a common route to DEEs (EuroEPINOMICS-RES Consortium, Epilepsy Phenome/Genome Project, Epi4K Consortium, 2014). Despite this, the relationship between the genetic causes of DEE, synaptic transmission, and seizures remain poorly understood. To address this first link for *DNM1*, we investigated how basic parameters of synaptic transmission are altered in a mouse model of *Dnm1* DEE at the four major connection types in the mammalian brain. Based on previous work in *Dnm1* KO and *Dnm1^Ftfl^* mice, we hypothesized that *Dnm1^Ftfl/Ftfl^* would disproportionately affect inhibitory synaptic transmission.

Although there were inhibitory transmission-specific effects, most of the synaptic changes caused by *Dnm1^Ftfl/Ftfl^* were not motif-specific. mPSC amplitude was increased regardless of presynaptic and postsynaptic neurotransmitter identity, and mPSC frequency was decreased. Because previous electron micrographs of *Dnm1* KO and *Dnm1^Ftfl/Ftfl^* synapses showed increased SV diameter, the larger amplitude of mEPSCs likely reflects the fusion of SVs that contain more neurotransmitter. We cannot rule out the possibility that postsynaptic receptor number is altered, possibly because of a deficit in receptor endocytosis, which all three dynamin isoforms have been implicated in (Carroll et al., 1999; Lu et al., 2007; Fiuza et al., 2017). However, shRNA-mediated knock-down of *Dnm3* was shown to reduce mEPSC amplitude because of impaired postsynaptic AMPA receptor recycling (Lu et al., 2007), suggesting altered postsynaptic endocytosis is an unlikely explanation for increased mPSC amplitude in *Dnm1^Ftfl/Ftfl^* cultures.

These EM studies also found a reduced total number of SVs at synaptic terminals. The overall reduction was only ∼20% in *Dnm1* KO (Ferguson et al., 2007), and slightly higher for *Dnm1^Ftfl^* (Dhindsa et al., 2015), although the number of docked SVs was not assessed. In accordance with this, we found mPSC frequency was reduced by 20–50% at *Dnm1^Ftfl/Ftfl^* synapses. Based on the assumption that the reduction in mPSC frequency we saw was caused by reduced SV number per terminal, we immunostained for synaptic markers on recorded, biocytin-filled neurons. We expected to find similar numbers of synapses and reduced mPSC frequency per synapse. Instead, we found reduced numbers of glutamatergic and GABAergic synapses, but unchanged mPSC frequency per synapse, suggesting that the primary driver of the reduced mPSC frequency was reduced synapse number and not reduced SV number per synapse. Reduced synapse number was not previously reported for *Dnm1* KO or *Dnm1/3* DKO cultures, however, conditional deletion of *Dnm1* and *Dnm3* in the Calyx of Held altered synapse maturation and decreased mEPSC frequency (Fan et al., 2016). Thus, our data suggest that *Dnm1^Ftfl/Ftfl^* causes a broad reduction in synapse formation or maintenance. One caveat to this interpretation is that some synapses with severely reduced SV number could fall below our detection limit, because we used the SV-associated proteins VGLUT1 and VGAT to mark synapses.

Although the changes in mPSCs and synapse number affected all motifs, evoked IPSCs onto glutamatergic neurons were specifically decreased. This observation is broadly consistent with the notion that altered inhibitory synaptic transmission underlies many DEEs (Bernard, 2012), and that expression of *Dnm1^Ftfl^* in inhibitory neurons of mice was sufficient to cause seizures and death (Asinof et al., 2015). Investigation into the physiological mechanism of the eIPSC decrease showed that the RRP of SVs was smaller at the I-E motif and that there was a deficit in recovery of the synaptic response after HFS. The reason for this motif specificity is unclear. The most straightforward possibility is that SV demand is greater at inhibitory synapses, as has been suggested previously (Hayashi et al., 2008). Because inhibitory neurons make strong synapses onto excitatory neurons and fire APs at relatively high tonic rates, they are naturally prone to SV depletion, as well as manipulations of endocytosis machinery (Hayashi et al., 2008). Further, because *Dnm1* function is activity dependent (Ferguson et al., 2007; Armbruster et al., 2013), inhibitory neurons may rely more heavily on *Dnm1* than comparatively less active excitatory neurons. However, this explanation fails to account for the fact that eIPSC amplitude at the I-I motif was unaffected.

An alternative explanation involves potential differences in the development of I-E and I-I synapses. Recent evidence suggests that excitatory activity drives amplification of inhibitory synapses onto excitatory neurons, both *in vivo* and *in vitro* (Xue et al., 2014). *In vitro*, this amplification can occur through increased synapse number in hippocampal interneurons (Chang et al., 2014), or increased quantal size and SV pool size in striatal interneurons (Paraskevopoulou et al., 2019). Pairwise comparisons of control motifs in our dataset found there are far more I-E than I-I synapses, and mean eIPSCs were larger at I-E than I-I synapses (although this did not reach statistical significance). This suggests that the I-E motif selectively amplifies through a combination of synapse number and SV pool size. Pairwise comparison of eIPSCs at *Dnm1^Ftfl/Ftfl^* I-E synapses to *Dnm1^Ftfl/Ftfl^* and control I-I synapses indicate they are similar. This observation suggests motif-specific effects of synaptic amplification fail to occur in *Dnm1^Ftfl/Ftfl^* neurons, and that normal *Dnm1* activity is necessary to facilitate the required increase in SV and synapse number. This may occur because of reduced excitatory transmission, and therefore reduced activity, or effects of the Fitful variant in regulating other amplification-related signaling, which is known to involve the BDNF/TrkB pathway (Marty et al., 2000; Paraskevopoulou et al., 2019).

One might expect that impairment of the I-E motif would lead to hyperactivity through disinhibition of excitatory neurons. While our calcium imaging observations showed increased fluorescence amplitudes and longer decay times, potentially indicative of increased spiking in *Dnm1^Ftfl/Ftfl^* neurons, these observations often do not directly translate into spiking activity. Indeed, cell-attached recordings revealed reduced spike frequency among excitatory neurons, suggesting that instead the calcium response per spike is altered in *Dnm1^Ftfl/Ftfl^* neurons, or altered spike timing within fluorescence events. This, along with our observations of reduced sPSC frequency, slower SLE onset, and reduced SLE duration, suggest the overall excitability of *Dnm1^Ftfl/Ftfl^* cultures is reduced. This was not attributable to changes in passive membrane properties or baseline neuronal excitability. Thus, other phenotypes we have observed may be responsible for depressing activity. For example, reduced overall connectivity, as suggested by reduced synapse number, may prevent percolation of activity through the network (Soriano et al., 2008). A previous imaging study of *Dnm1/3* DKO neurons found severely reduced event frequency (Lou et al., 2012); thus, the *Dnm1^Ftfl/Ftfl^* phenotype may represent a milder version of the *Dnm1/3* DKO. Other *in vitro* networks of neurons from DEE-causing gene variants, including *Scn1a* (Hedrich et al., 2014), *GNB1* (Colombo et al., 2019), *Kcnt1* (Shore et al., 2020), and *Pten* (Barrows et al., 2017), have shown increases in activity and/or synchrony, as measured with Ca^2+^ imaging or multielectrode arrays. Our results suggest, however, that in *Dnm1^Ftfl/Ftfl^* networks at least, higher order levels of brain architecture are necessary to generate epileptic activity. Spike-and-wave events, as well as absence seizures, are known to depend on dysfunction within the thalamocortical circuit (McCormick and Contreras, 2001; Maheshwari and Noebels, 2014). *Dnm1^Ftfl/Ftfl^* mice have prominent spike-and-wave events, and both forms of seizures have been reported in children with *DNM1* encephalopathy (von Spiczak et al., 2017).

Although overall levels of neural activity in *Dnm1^Ftfl/Ftfl^* cultures were comparable to controls or lower, *Dnm1^Ftfl/Ftfl^* neurons degenerated between DIV12 and DIV21. Neuronal degeneration is a feature of many epilepsies, both genetic and acquired, and is often considered a result of hyperactivity or excitotoxicity (Olney et al., 1986; Meldrum, 1993; Rakhade and Jensen, 2009). However, our observations that spontaneous AP firing is lower in *Dnm1*^Ftfl^ excitatory neurons, and that AP ISIs are longer in both neuron types, suggest that neuronal degeneration in *Dnm1^Ftfl/Ftfl^* mice occurs independent of seizures or hyperactivity. It is possible that the degeneration is exacerbated *in vitro*; however, recent *in vivo* work in *Dnm1^Ftfl/Ftfl^* mice also found increased neuronal degeneration, which was ameliorated by knock-down of *Dnm1^Ftfl^* (Aimiuwu et al., 2020). Notably, neuronal degeneration in both *Dnm1^Ftfl/Ftfl^* mice and cultures are present after PND or DIV14, which coincides with the onset of lethality, but is later than the appearance of seizures (Boumil et al., 2010; Asinof et al., 2015).

The mechanism of degeneration in *Dnm1^Ftfl/Ftfl^* neurons is unclear, but may involve altered intracellular signaling. DNM1 was previously shown to facilitate TrkA (Bodmer et al., 2011), TrkB (Liu et al., 2015), and EGFR endocytosis (Lee et al., 2006; Chen et al., 2017), suggesting it is important for regulation of growth factor signaling. Additionally, recent evidence has implicated DNM1 in endocytosis of the TNF-related apoptosis inducing ligand–death receptor (Reis et al., 2017). While this evidence comes from cancer cell lines, it demonstrates that DNM1 may be directly involved in suppressing pro-apoptotic signaling. An important caveat is that simple loss of *Dnm1* in neurons does not induce degeneration (Ferguson et al., 2007), which suggests that other dynamin isoforms can compensate in neurons, and that *Dnm1^Ftfl^* interferes with the function of other dynamin isoforms. In support of this *Dnm1/2/3* TKO neurons have also been reported to not be viable (Wu et al., 2014). Excitotoxicity, even without increased neural activity, is also a possibility, as *Dnm1*^Ftfl^ mEPSCs were larger and slower to decay, which could lead to pathologic Ca^2+^ entry through NMDA receptors.

In summary, the work we present here provides a more detailed picture of synaptic and network dysfunction in the context of the seizure-causing *Dnm1^Ftfl^* variant. Consistent with ubiquitous neuronal expression of the *Dnm1* gene, we have observed similar phenotypes across all synaptic motifs, including increased mPSC size and decreased synapse number. However, we also observe a motif-specific reduction in evoked synaptic strength of inhibitory neurons onto excitatory neurons, suggesting inhibitory restraint of network activity is selectively impaired. Despite this, we see reduced activity in *Dnm1^Ftfl/Ftfl^* networks *in vitro*, suggesting higher order brain architecture is necessary to produce seizures in *Dnm1^Ftfl/Ftfl^* mice. Finally, we see neuronal degeneration in *Dnm1^Ftfl/Ftfl^* cultures, suggesting the degeneration seen *in vivo* is not exclusively because of neural hyperactivity. Future work will be necessary to determine the mechanism of motif specificity, as well as to characterize the process leading to neuronal degeneration.

## Synthesis

Reviewing Editor: Quentin Pittman, University of Calgary Decisions are customarily a result of the Reviewing Editor and the peer reviewers coming together and discussing their recommendations until a consensus is reached. When revisions are invited, a fact-based synthesis statement explaining their decision and outlining what is needed to prepare a revision will be listed below. The following reviewer(s) agreed to reveal their identity: Ruchi Malik, Zhong-Wei Zhang.

Dmn-1 gene is linked with developmental epileptic encephalopathies (DEE). Mouse models expressing Dmn-1 gene mutations (such as Fitful) replicate key aspects of these disorders and therefore have been instrumental in understanding the underlying neuronal deficits. Dynamin1 is a key regulator of synaptic vesicle endocytosis; previous studies in the mouse have shown that deletion of Dnm1 disrupts synaptic transmission. However, the effects of Dnm1 mutations on synaptic transmission are not well defined. This knowledge gap was addressed by the authors in this study using a mouse model of Dnm1-associated DEE.

The present study characterizes how the expression of a dominant-negative variant of Dnm1, a gene associated with developmental epileptic encephalopathies, affects excitatory and inhibitory synapses in cultured cortical neurons. The authors describe the changes in both excitatory and inhibitory synaptic transmission in cortical neurons expressing Fitful variant of Dmn-1 gene. The primary conclusion is that the expression of Fitful variant of Dnm1 gene affects spontaneous synaptic transmission at both excitatory and inhibitory synapses. The authors, however, find synapse-specific deficits in evoked transmission at inhibitory synapses on excitatory neurons. This was largely due to a reduction in readily releasable vesicle pool at these synapses. In addition to the synaptic changes, Fitful expression affected neuronal health of cortical cultured neurons in this study. Overall, this is a well-constructed study that extends our knowledge of the role of Dynamin 1 in synaptic transmission and the data presented support the conclusions.

Comments:

A.Previous studies have shown that the Dnm1 variant causes DEE and neurodegeneration in mice. In this study, the authors suggest that neurodegeneration may be independent of seizures and excitotoxicity. This point has important implications for the treatment of DEEs. However, stronger evidence is needed. For the following 3 reasons, it is suggested that you quantify burst firing in the mutant neurons:

1.The first piece of evidence presented in the manuscript came from calcium imaging. The data showed that there was an increase in the amplitude of Ca2+ transients in mutant neurons at DIV 7-11, but the effect was not significant at DIV12-16 or DIV17-21 (Figure 5). And the frequency of the event was different between mutant and control across developmental epochs. Based the data presented in Fig. 5C, however, the events from mutant neurons seem to have longer duration than those in control neurons at DIV12-16. If this is true, it could mean that mutant neurons fire more bursts of action potentials than control neurons.

2. The second piece of evidence came from spontaneous synaptic events. The data showed that the frequency of both sIPSCs and sEPSCs decreased in mutant neurons (Figure 6). However, sPSCs are the mixture of mPSCs and PSCs induced by action potentials. It is unclear in this case, what percentage of sPSCs belongs to those induced by action potentials. One way to ascertain that neuronal activity is not affected by the variant would be to record action potentials from these neurons. Loose patch recording can be used to avoid potential complications associated with whole-cell recording. This will also tell us whether there are more bursts in mutant neurons.

3. There is an overriding question as to the relationship between seizure like events and neuronal degeneration. The last piece of evidence came from seizure-like events induced by the application of GABAA blocker bicuculline. Notably, seizure-like events were much shorter in duration in mutant neurons (Figure 7B2). However, the significance of this result is not entirely clear because we don't know what causes the seizure-like events to stop. Alternatively, chronic blockade of AMPA and NMDA receptors may be used to test whether neurodegeneration found in mutant cultures is due to excitotoxicity.

B.The reviewers are curious if the reduction in synapse number/density is different along the soma-dendritic axis in the Synapse number analysis in Fig. 2. It is possible that the relative reduction in perisomatic synapses vs. synapses located more distally might be different in Fitful neurons. Since the currents originating from perisomatic synapses are often larger in voltage-clamp recordings, the suggested analysis of proximal vs distal synapses may also shed some light on why mPSC amplitudes are larger in Fitful neurons.

C.Based on previous studies, deficits in inhibitory neurons in Fitful mice are thought to be the underlying cause of seizures and death. Therefore, the inhibitory neurons expressing Fitful may be more susceptible to neuronal degeneration. To address this possibility, it would be interesting if the analysis in Fig. 8 can be extended to compare changes in survival/degeneration between excitatory and inhibitory neurons.

Minor point-

Scale bar is missing in Fig. 2A

## Author Response

Dnm1 Reviewer Response

We thank the reviewers for their time reading the manuscript, insightful comments, and clear guidance. We have revised the manuscript with new experiments, analysis, and rewriting which we believe addresses all of the reviewer concerns and substantially improves the manuscript.

A. “Previous studies have shown that the Dnm1 variant causes DEE and neurodegeneration in mice. In this study, the authors suggest that neurodegeneration may be independent of seizures and excitotoxicity. This point has important implications for the treatment of DEEs. However, stronger evidence is needed. For the following 3 reasons, it is suggested that you quantify burst firing in the mutant neurons:

Based the data presented in Fig. 5C... the events from mutant neurons seem to have longer duration than those in control neurons at DIV12-16. If this is true, it could mean that mutant neurons fire more bursts of action potentials than control neurons.”

We first analyzed the decay time of the fluorescence events, as the reviewers astutely point out that the decay time appears longer. The results of this analysis showed that the 20%-80% decay time was indeed longer in Dnm1Ftfl neurons, in the two earlier developmental epochs. This new analysis is now included in a revised Figure 6.

"One way to ascertain that neuronal activity is not affected by the variant would be to record action potentials from these neurons. Loose patch recording can be used to avoid potential complications associated with whole-cell recording. This will also tell us whether there are more bursts in mutant neurons.”

To test assay neural activity with better temporal resolution and determine whether increased bursting could account for the slowing of the fluorescence decay in Dnm1Ftfl neurons, we performed cell-attached recordings, as suggested by the reviewers, on both excitatory and inhibitory neurons, as marked by CaMKII or Dlx5/6 promoter driven fluorescence. The results of this experiments are presented in a new Figure 8. We found that Dnm1Ftfl excitatory neurons fire fewer APs overall, as evidenced by a reduced mean firing rate and increased median interspike interval. Despite the overall decrease in AP frequency, however, it is still possible that Dnm1Ftfl excitatory neurons fire more APs in bursts. To examine this, we plotted the relative frequency of interspike intervals on a histogram. Examination of the histogram (new Figure 8C) shows a decrease in very short latency ISIs in Dnm1Ftfl neurons, suggesting that they are not bursting more. There was a small increase in the relative frequency of ISIs around 100 ms, which we believe may contribute to the slower decay of fluorescence in Dnm1Ftfl excitatory neurons. However, altered Ca2+ dynamics are also highly possible, and we have discussed this alternative hypothesis.

"There is an overriding question as to the relationship between seizure like events and neuronal degeneration. . .Notably, seizure-like events were much shorter in duration in mutant neurons (Figure 7B2). However, the significance of this result is not entirely clear because we don’t know what causes the seizure-like events to stop. Alternatively, chronic blockade of AMPA and NMDA receptors may be used to test whether neurodegeneration found in mutant cultures is due to excitotoxicity.”

We agree with the reviewers that the relationship between the shorter duration of the SLEs and the degeneration is not entirely clear, as we do not know what causes them to stop, and they are artificially induced. However, with the new data showing that AP firing is reduced in Dnm1Ftfl cultures, we believe we can put the result in a better context, i.e. even if a hyperactive state is induced in Dnm1Ftfl cultures, it is not longer lasting or more intense.

We did not try chronic blockade of glutamate receptors. it is possible that excitotoxicity is playing a role, as both the amplitude and decay time of mEPSCs are increased, but that this is independent of any change in neural activity (AP firing). We omitted references to excitotoxicity in the manuscript, added this caveat to the discussion, and instead focused on neural degeneration in the absence of hyperactivity as the stronger conclusion.

B. “The reviewers are curious if the reduction in synapse number/density is different along the soma-dendritic axis in the Synapse number analysis in Fig. 2. It is possible that the relative reduction in perisomatic synapses vs. synapses located more distally might be different in Fitful neurons. Since the currents originating from perisomatic synapses are often larger in voltage-clamp recordings, the suggested analysis of proximal vs distal synapses may also shed some light on why mPSC amplitudes are larger in Fitful neurons.”

We appreciate the insightful suggestion of the reviewers. To address this, we went back and counted the fraction of synapses that were made onto the somatic compartment in WT and Dnm1Ftfl neurons. The results of this new analysis are in a revised Figure 2. We did not detect a difference in the fraction of somatic/dendritic synapses, suggesting that the decrease occurs in both compartments. Also, although we agree that perisomatic synapses would tend to be larger amplitude in voltage-clamp recordings, they would also tend to have a shorter decay time, which is the opposite of what we observe in Dnm1Ftfl neurons.

C. “Based on previous studies, deficits in inhibitory neurons in Fitful mice are thought to be the underlying cause of seizures and death. Therefore, the inhibitory neurons expressing Fitful may be more susceptible to neuronal degeneration. To address this possibility, it would be interesting if the analysis in Fig. 8 can be extended to compare changes in survival/degeneration between excitatory and inhibitory neurons.”

This is another good point. To address it, we labeled excitatory and inhibitory neurons with two AAVs that express eGFP or mCherry under control of the CaMKII or Dlx5/6 promoter, and then counted them at three time points. The results of this new experiment are presented in a new Figure 10. We found that the density of inhibitory neurons was slightly lower at all time points in Dnm1Ftfl. Excitatory neurons were equal in density at the early time point, but had declined sharply but the final time point. Our interpretation of this data is that Dnm1Ftfl causes a small reduction in the overall numbers of inhibitory neurons, but that the widespread neuronal death we observed during week two in vitro is due to loss of large numbers of excitatory neurons.
