# Supplementary material for: Altered Fast Synaptic Transmission in a Mouse Model of DNM1-Associated Developmental Epileptic Encephalopathy
Source: eNeuro. 2021 Mar 9;8(2):ENEURO.0269-20.2020. doi: 10.1523/ENEURO.0269-20.2020 (PMC7986544; doi:10.1523/ENEURO.0269-20.2020)
Supplement: Extended Data Figure 1-1 — mPSC pairwise comparisons Download Figure 1-1, DOCX file. [file enu-eN-NWR-0269-20-s03.docx]

| **Figure 1-1 - mPSC Pairwise Comparisons** | | | | | | |
| --- | --- | --- | --- | --- | --- | --- |
| **Comparison** | | | **Mean Difference** | **P-value** | **95% Wald Confidence Interval for Difference** | |
|  |  |  |  |  | **Lower** | **Upper** |
| **Frequency** | **Ftfl I-I** | **WT I-I** | -0.83 | 0.008 | -1.45 | -0.22 |
|  | **Ftfl I-E** | **WT I-E** | -0.11 | 0.109 | -0.24 | 0.02 |
|  | **Ftfl E-I** | **WT E-I** | -4.60 | 0.017 | -8.37 | -0.84 |
|  | **Ftfl E-E** | **WT E-E** | -0.53 | 0.002 | -0.86 | -0.19 |
| **Amplitude** | **Ftfl I-I** | **WT I-I** | 12.25 | 0.003 | 4.03 | 20.46 |
|  | **Ftfl I-E** | **WT I-E** | 7.64 | 0.031 | 0.70 | 14.58 |
|  | **Ftfl E-I** | **WT E-I** | 5.37 | 0.039 | 0.26 | 10.47 |
|  | **Ftfl E-E** | **WT E-E** | 2.40 | 0.064 | -0.14 | 4.95 |
| **Decay** | **Ftfl I-I** | **WT I-I** | 5.91 | 0.078 | -0.67 | 12.48 |
|  | **Ftfl I-E** | **WT I-E** | 6.39 | 0.008 | 1.65 | 11.12 |
|  | **Ftfl E-I** | **WT E-I** | 0.11 | 0.789 | -0.70 | 0.92 |
|  | **Ftfl E-E** | **WT E-E** | 0.21 | 0.345 | -0.23 | 0.66 |
| Mean differences, p-values, and confidence intervals were derived from comparison of estimated marginal means from generalized estimating equations. | | | | | | |
