# Supplementary material for: Altered Fast Synaptic Transmission in a Mouse Model of DNM1-Associated Developmental Epileptic Encephalopathy
Source: eNeuro. 2021 Mar 9;8(2):ENEURO.0269-20.2020. doi: 10.1523/ENEURO.0269-20.2020 (PMC7986544; doi:10.1523/ENEURO.0269-20.2020)
Supplement: Extended Data Figure 2-1 — Synapse counts, density, and mPSC frequency Download Figure 2-1, DOCX file. [file enu-eN-NWR-0269-20-s04.docx]

| **Figure 2-1 - Synapse Counts, Density, and mPSC frequency** | | | | | | |
| --- | --- | --- | --- | --- | --- | --- |
| **Comparison** | | | **Mean Difference** | **P-value** | **95% Wald Confidence Interval for Difference** | |
|  |  |  |  |  | **Lower** | **Upper** |
| **Counts** | **Ftfl I-I** | **WT I-I** | -42.50 | 0.171 | -103.34 | 18.33 |
|  | **Ftfl I-E** | **WT I-E** | -237.40 | 0.256 | -647.14 | 172.34 |
|  | **Ftfl E-I** | **WT E-I** | -163.22 | 0.055 | -329.76 | 3.32 |
|  | **Ftfl E-E** | **WT E-E** | -263.38 | 0.051 | -527.57 | 0.82 |
| **Density** | **Ftfl I-I** | **WT I-I** | -0.016 | 0.022 | -0.030 | -0.002 |
|  | **Ftfl I-E** | **WT I-E** | -0.065 | 0.055 | -0.131 | 0.001 |
|  | **Ftfl E-I** | **WT E-I** | -0.056 | 0.001 | -0.089 | -.022 |
|  | **Ftfl E-E** | **WT E-E** | -0.090 | <0.001 | -0.134 | -0.047 |
| **Somatic Synapse Counts** | **Ftfl I-I** | **WT I-I** | 0.019 | 0.803 | -0.128 | 0.165 |
|  | **Ftfl I-E** | **WT I-E** | 0.000 | 0.994 | -0.034 | 0.034 |
|  | **Ftfl E-I** | **WT E-I** | 0.069 | 0.337 | -0.072 | 0.209 |
|  | **Ftfl E-E** | **WT E-E** | 0.012 | 0.485 | -0.022 | 0.047 |
| **mPSC Frequency per Synapse (mHz)** | **Ftfl I-I** | **WT I-I** | 2.46 | .614 | -7.09 | 12.00 |
|  | **Ftfl I-E** | **WT I-E** | 0.182 | .443 | -0.283 | 0.647 |
|  | **Ftfl E-I** | **WT E-I** | 6.96 | .619 | -20.50 | 34.42 |
|  | **Ftfl E-E** | **WT E-E** | 2.07 | .069 | -0.163 | 4.30 |
| Mean differences, p-values, and confidence intervals were derived from comparison of estimated marginal means from generalized estimating equations. | | | | | | |
