# Supplementary material for: Altered Fast Synaptic Transmission in a Mouse Model of DNM1-Associated Developmental Epileptic Encephalopathy
Source: eNeuro. 2021 Mar 9;8(2):ENEURO.0269-20.2020. doi: 10.1523/ENEURO.0269-20.2020 (PMC7986544; doi:10.1523/ENEURO.0269-20.2020)
Supplement: Extended Data Figure 3-1 — ePSC pairwise comparisons Download Figure 3-1, DOCX file. [file enu-eN-NWR-0269-20-s05.docx]

| **Figure 3-1 - Evoked PSC Pairwise Comparisons** | | | | | |
| --- | --- | --- | --- | --- | --- |
| **Comparison** | | **Mean Difference** | **P-value** | **95% Wald Confidence Interval for Difference** | |
|  |  |  |  | **Lower** | **Upper** |
| **Ftfl I-I** | **WT I-I** | -132.71 ± 328.9 | .687 | -777.3489 | 511.9327 |
| **Ftfl I-E** | **WT I-E** | -1212.91 ± 453.41 | .007 | -2101.5665 | -324.2512 |
| **Ftfl E-I** | **WT E-I** | -132.50 ± 161.51 | .412 | -449.0515 | 184.0435 |
| **Ftfl E-E** | **WT E-E** | 55.10 ± 95.18 | .563 | -131.4470 | 241.6560 |
| Mean differences, p-values, and confidence intervals were derived from comparison of estimated marginal means from generalized estimating equations. | | | | | |
