# Supplementary material for: Altered Fast Synaptic Transmission in a Mouse Model of DNM1-Associated Developmental Epileptic Encephalopathy
Source: eNeuro. 2021 Mar 9;8(2):ENEURO.0269-20.2020. doi: 10.1523/ENEURO.0269-20.2020 (PMC7986544; doi:10.1523/ENEURO.0269-20.2020)
Supplement: Extended Data Figure 4-2 — Synaptic dynamics pairwise comparisons Download Figure 4-2, DOCX file. [file enu-eN-NWR-0269-20-s07.docx]

| **Figure 4-2 - Synaptic Dynamics Pairwise Comparisons** | | | | | | |
| --- | --- | --- | --- | --- | --- | --- |
|  | **Comparison** | | **Mean Difference** | **P-value** | **95% Wald Confidence Interval for Difference** | |
|  |  |  |  |  | **Lower** | **Upper** |
| **Tetanic Depression** | **Ftfl** | **WT** | 0.019 | 0.373 | -0.023 | 0.06 |
| **Tetanic Recovery** | **Ftfl** | **WT** | -0.24 | <0.001 | -0.27 | -0.22 |
| **10 Hz Depression** | **Ftfl** | **WT** | -0.015 | 0.866 | -0.187 | 0.158 |
| **Paired-Pulse Ratio** | **Ftfl** | **WT** | -0.0026 | 0.780 | -0.0206 | 0.0155 |
| Mean differences, p-values, and confidence intervals were derived from comparison of estimated marginal means from generalized estimating equations. | | | | | | |
