# Supplementary material for: Altered Fast Synaptic Transmission in a Mouse Model of DNM1-Associated Developmental Epileptic Encephalopathy
Source: eNeuro. 2021 Mar 9;8(2):ENEURO.0269-20.2020. doi: 10.1523/ENEURO.0269-20.2020 (PMC7986544; doi:10.1523/ENEURO.0269-20.2020)
Supplement: Extended Data Figure 5-1 — Calcium imaging model effects Download Figure 5-1, DOCX file. [file enu-eN-NWR-0269-20-s08.docx]

| **Figure 5-1 Calcium Imaging Model Effects** | | | | | | |
| --- | --- | --- | --- | --- | --- | --- |
| **Factor** | **Event Count** | | **Peak Fluorescence** | | **80/20 Decay Time** | |
|  | **Wald Chi-Square** | **P-value** | **Wald Chi-Square** | **P-value** | **Wald Chi-Square** | **P-value** |
| **Intercept** | 831.815 | <0.001 | 709.177 | <0.001 | 71.789 | <0.001 |
| **Genotype** | .219 | 0.639 | 1.103 | 0.294 | 3.22 | 0.073 |
| **Epoch** | 124.733 | <0.001 | 132.581 | <0.001 | 275.978 | <0.001 |
| **Genotype x Epoch** | 8.292 | 0.016 | 2.064 | 0.356 | 11.738 | 0.003 |
| Tests of model effects for genotype and developmental epoch on number of calcium events and peak fluorescence. Wald Chi-squares and P-values were obtained using generalized estimating equations. | | | | | | |
