# Supplementary material for: Altered Fast Synaptic Transmission in a Mouse Model of DNM1-Associated Developmental Epileptic Encephalopathy
Source: eNeuro. 2021 Mar 9;8(2):ENEURO.0269-20.2020. doi: 10.1523/ENEURO.0269-20.2020 (PMC7986544; doi:10.1523/ENEURO.0269-20.2020)
Supplement: Extended Data Figure 5-2 — Calcium imaging pairwise comparisons Download Figure 5-2, DOCX file. [file enu-eN-NWR-0269-20-s09.docx]

| **Figure 5-2 - Calcium Imaging Pairwise Comparisons** | | | | | | |
| --- | --- | --- | --- | --- | --- | --- |
|  | **Comparison** | | **Mean Difference** | **P-value** | **95% Wald Confidence Interval for Difference** | |
|  |  |  |  |  | **Lower** | **Upper** |
| **Event Count** | **Ftfl Epoch 1** | **WT Epoch 1** | -0.855 | 0.066 | -1.765 | .055 |
|  | **Ftfl Epoch 2** | **WT Epoch 2** | 0.861 | 0.161 | -.344 | 2.066 |
|  | **Ftfl Epoch 3** | **WT Epoch 3** | -3.950 | 0.612 | -19.228 | 11.328 |
| **Peak Fluorescence** | **Ftfl Epoch 1** | **WT Epoch 1** | 0.0129 | <0.001 | 0.0106 | 0.0152 |
|  | **Ftfl Epoch 2** | **WT Epoch 2** | 0.0288 | 0.367 | -0.0338 | 0.0913 |
|  | **Ftfl Epoch 3** | **WT Epoch 3** | -0.0114 | 0.634 | -0.0583 | 0.0355 |
| **80/20 Decay Time** | **Ftfl Epoch 1** | **WT Epoch 1** | 0.185 | 0.001 | 0.072 | 0.299 |
|  | **Ftfl Epoch 2** | **WT Epoch 2** | 0.605 | 0.001 | 0.261 | 0.949 |
|  | **Ftfl Epoch 3** | **WT Epoch 3** | 0.303 | 0.557 | -0.709 | 1.316 |
| Mean differences, p-values, and confidence intervals were derived from comparison of estimated marginal means from generalized estimating equations. | | | | | | |
