# Supplementary material for: Altered Fast Synaptic Transmission in a Mouse Model of DNM1-Associated Developmental Epileptic Encephalopathy
Source: eNeuro. 2021 Mar 9;8(2):ENEURO.0269-20.2020. doi: 10.1523/ENEURO.0269-20.2020 (PMC7986544; doi:10.1523/ENEURO.0269-20.2020)
Supplement: Extended Data Figure 6-1 — sPSC pairwise comparisons Download Figure 6-1, DOCX file. [file enu-eN-NWR-0269-20-s10.docx]

| **Figure 6-1 - sPSC Pairwise Comparisons** | | | | | | |
| --- | --- | --- | --- | --- | --- | --- |
|  | | | | | | |
| **Comparison** | | | **Mean Difference** | **P-value** | **95% Wald Confidence Interval for Difference** | |
|  |  |  |  |  | **Lower** | **Upper** |
| **Frequency** | **Ftfl I-I** | **WT I-I** | -1.91 | 0.026 | -3.59 | -0.23 |
|  | **Ftfl I-E** | **WT I-E** | -2.34 | 0.001 | -3.69 | -1.00 |
|  | **Ftfl E-I** | **WT E-I** | -12.73 | <0.001 | -19.12 | -6.34 |
|  | **Ftfl E-E** | **WT E-E** | -9.57 | 0.001 | -15.12 | -4.02 |
| **Charge** | **Ftfl I-I** | **WT I-I** | 318.50 | 0.002 | 121.44 | 515.56 |
|  | **Ftfl I-E** | **WT I-E** | 329.48 | 0.002 | 115.97 | 542.98 |
|  | **Ftfl E-I** | **WT E-I** | 15.83 | 0.280 | -12.91 | 44.56 |
|  | **Ftfl E-E** | **WT E-E** | 35.22 | 0.024 | 4.59 | 65.85 |
| Mean differences, p-values, and confidence intervals were derived from comparison of estimated marginal means from generalized estimating equations. | | | | | | |
