# Supplementary material for: Altered Fast Synaptic Transmission in a Mouse Model of DNM1-Associated Developmental Epileptic Encephalopathy
Source: eNeuro. 2021 Mar 9;8(2):ENEURO.0269-20.2020. doi: 10.1523/ENEURO.0269-20.2020 (PMC7986544; doi:10.1523/ENEURO.0269-20.2020)
Supplement: Extended Data Figure 7-1 — Cell attached pairwise comparisons Download Figure 7-1, DOCX file. [file enu-eN-NWR-0269-20-s11.docx]

| **Figure 7-1 -Cell Attached Pairwise Comparisons** | | | | | | |
| --- | --- | --- | --- | --- | --- | --- |
|  | | | | | | |
| **Comparison** | | | **Mean Difference** | **P-value** | **95% Wald Confidence Interval for Difference** | |
|  |  |  |  |  | **Lower** | **Upper** |
| **Mean Firing Rate** | **Ftfl E** | **WT E** | -0.61 | 0.003 | -1.02 | -0.21 |
|  | **Ftfl I** | **WT I** | 0.36 | 0.87 | -3.97 | 4.69 |
| **Interspike interval** | **Ftfl E** | **WT E** | 2.08 | 0.048 | 1.28 | 5.22 |
|  | **Ftfl I** | **WT I** | 0.07 | 0.24 | -0.05 | 0.19 |
| **Interspike interval CV** | **Ftfl E** | **WT E** | 2.93 | 0.88 | -34.6 | 40.5 |
|  | **Ftfl I** | **WT I** | -72.7 | 0.0004 | -113.0 | -32.3 |
| Mean differences, p-values, and confidence intervals were derived from comparison of estimated marginal means from generalized estimating equations. | | | | | | |
