# Supplementary material for: Altered Fast Synaptic Transmission in a Mouse Model of DNM1-Associated Developmental Epileptic Encephalopathy
Source: eNeuro. 2021 Mar 9;8(2):ENEURO.0269-20.2020. doi: 10.1523/ENEURO.0269-20.2020 (PMC7986544; doi:10.1523/ENEURO.0269-20.2020)
Supplement: Extended Data Figure 8-1 — SLE pairwise comparisons Download Figure 8-1, DOCX file. [file enu-eN-NWR-0269-20-s12.docx]

| **Figure 8-1 - Seizure-Like Event Pairwise Comparisons** | | | | | | |
| --- | --- | --- | --- | --- | --- | --- |
|  | **Comparison** | | **Mean Difference** | **P-value** | **95% Wald Confidence Interval for Difference** | |
|  |  |  |  |  | **Lower** | **Upper** |
| **Latency to Event (Seconds)** | **Ftfl** | **WT** | 7.35 | <0.001 | 4.96 | 10.21 |
| **Event Duration (Seconds)** | **Ftfl** | **WT** | -13.27 | <0.001 | -19.73 | -6.81 |
| **Event Charge (μA*ms)** | **Ftfl** | **WT** | -4.46 | 0.002 | -7.32 | -1.59 |
| Mean differences, p-values, and confidence intervals were derived from comparison of estimated marginal means from generalized estimating equations. | | | | | | |
