# Supplementary material for: Altered Fast Synaptic Transmission in a Mouse Model of DNM1-Associated Developmental Epileptic Encephalopathy
Source: eNeuro. 2021 Mar 9;8(2):ENEURO.0269-20.2020. doi: 10.1523/ENEURO.0269-20.2020 (PMC7986544; doi:10.1523/ENEURO.0269-20.2020)
Supplement: Extended Data Figure 9-1 — Cell death model effects Download Figure 9-1, DOCX file. [file enu-eN-NWR-0269-20-s01.docx]

| **Factor** | **Inhibitory neuron density** | | **Excitatory neuron density** | |
| --- | --- | --- | --- | --- |
|  | **Wald Chi-Square** | **P-value** | **Wald Chi-Square** | **P-value** |
| **Intercept** | 16814.762 | <0.001 | 2433.525 | <0.001 |
| **Genotype** | 28.508 | <0.001 | 7.689 | 0.006 |
| **DIV** | 8.408 | 0.015 | 76.045 | <0.001 |
| **Genotype x DIV** | 1.170 | 0.56 | 57.115 | <0.001 |

**Figure 10-1 Cell Counts Model Effects**

Tests of model effects for genotype and day *in vitro* on cell death. Wald Chi-squares and P-values were obtained using generalized estimating equations.
