# Supplementary material for: Altered Fast Synaptic Transmission in a Mouse Model of DNM1-Associated Developmental Epileptic Encephalopathy
Source: eNeuro. 2021 Mar 9;8(2):ENEURO.0269-20.2020. doi: 10.1523/ENEURO.0269-20.2020 (PMC7986544; doi:10.1523/ENEURO.0269-20.2020)
Supplement: Extended Data Figure 9-2 — Cell death pairwise comparisons Download Figure 9-2, DOCX file. [file enu-eN-NWR-0269-20-s02.docx]

| **Figure 9-2 Cell Death Pairwise Comparisons** | | | | | | |
| --- | --- | --- | --- | --- | --- | --- |
|  | **Comparison** | | **Mean Difference** | **P-value** | **95% Wald Confidence Interval for Difference** | |
|  |  |  |  |  | **Lower** | **Upper** |
| **Cell Death** | **Ftfl**  **DIV17** | **WT**  **DIV17** | 7.38 | 0.416 | -10.41 | 25.17 |
|  | **Ftfl**  **DIV17** | **Ftfl**  **DIV21** | -57.38 | 0.01 | -100.97 | -13.79 |
|  | **WT**  **DIV17** | **WT**  **DIV21** | 12.16 | 0.107 | -2.63 | 26.94 |
|  | **Ftfl**  **DIV21** | **WT**  **DIV21** | 76.92 | 0.003 | 26.32 | 127.53 |
| Mean differences, p-values, and confidence intervals were derived from comparison of estimated marginal means from generalized estimating equations. | | | | | | |
